# Systemic complement activation is associated with respiratory failure in COVID-19 hospitalized patients

**DOI:** 10.1073/pnas.2010540117

**Published:** 2020-09-17

**Authors:** Jan C. Holter, Soeren E. Pischke, Eline de Boer, Andreas Lind, Synne Jenum, Aleksander R. Holten, Kristian Tonby, Andreas Barratt-Due, Marina Sokolova, Camilla Schjalm, Viktoriia Chaban, Anette Kolderup, Trung Tran, Torleif Tollefsrud Gjølberg, Linda G. Skeie, Liv Hesstvedt, Vidar Ormåsen, Børre Fevang, Cathrine Austad, Karl Erik Müller, Cathrine Fladeby, Mona Holberg-Petersen, Bente Halvorsen, Fredrik Müller, Pål Aukrust, Susanne Dudman, Thor Ueland, Jan Terje Andersen, Fridtjof Lund-Johansen, Lars Heggelund, Anne M. Dyrhol-Riise, Tom E. Mollnes

**Affiliations:** ^a^Department of Microbiology, Oslo University Hospital, 0424 Oslo, Norway;; ^b^Institute of Clinical Medicine, University of Oslo, 0315 Oslo, Norway;; ^c^Division of Emergencies and Critical Care, Oslo University Hospital, 0424 Oslo, Norway;; ^d^Department of Immunology, Oslo University Hospital, 0424 Oslo, Norway;; ^e^Department of Infectious Diseases, Oslo University Hospital, 0424 Oslo, Norway;; ^f^Department of Acute Medicine, Oslo University Hospital, 0424 Oslo, Norway;; ^g^Department of Pharmacology, University of Oslo, 0315 Oslo, Norway;; ^h^Department of Ophthalmology, Oslo University Hospital, 0424 Oslo, Norway;; ^i^Research Institute of Internal Medicine, Oslo University Hospital, 0424 Oslo, Norway;; ^j^Section of Clinical Immunology and Infectious Diseases, Oslo University Hospital, 0424 Oslo, Norway;; ^k^Department of Internal Medicine, Vestre Viken Hospital Trust, 3004 Drammen, Norway;; ^l^Department of Clinical Science, Faculty of Medicine, University of Bergen, 5007 Bergen, Norway;; ^m^Faculty of Health Sciences, K.G. Jebsen Thrombosis Research and Expertise Center, University of Tromsø, 9037 Tromsø, Norway;; ^n^ImmunoLingo Convergence Centre, University of Oslo, 0315 Oslo, Norway;; ^o^Research Laboratory, Nordland Hospital Bodø, 8092 Bodø, Norway;; ^p^Centre of Molecular Inflammation Research, Norwegian University of Science and Technology, 7491 Trondheim, Norway

**Keywords:** complement system, COVID-19, SARS-CoV-2, sC5b-9, respiratory failure

## Abstract

Respiratory failure in the acute respiratory syndrome coronavirus 2 (SARS-CoV-2) pandemic is hypothesized to be driven by an overreacting innate immune response, where the complement system is a key player. In this prospective cohort study of 39 hospitalized coronavirus disease COVID-19 patients, we describe systemic complement activation and its association with development of respiratory failure. Clinical data and biological samples were obtained at admission, days 3 to 5, and days 7 to 10. Respiratory failure was defined as PO_2_/FiO_2_ ratio of ≤40 kPa. Complement activation products covering the classical/lectin (C4d), alternative (C3bBbP) and common pathway (C3bc, C5a, and sC5b-9), the lectin pathway recognition molecule MBL, and antibody serology were analyzed by enzyme-immunoassays; viral load by PCR. Controls comprised healthy blood donors. Consistently increased systemic complement activation was observed in the majority of COVID-19 patients during hospital stay. At admission, sC5b-9 and C4d were significantly higher in patients with than without respiratory failure (*P *= 0.008 and *P* = 0.034). Logistic regression showed increasing odds of respiratory failure with sC5b-9 (odds ratio 31.9, 95% CI 1.4 to 746, *P* = 0.03) and need for oxygen therapy with C4d (11.7, 1.1 to 130, *P* = 0.045). Admission sC5b-9 and C4d correlated significantly to ferritin (*r* = 0.64, *P* < 0.001; *r* = 0.69, *P* < 0.001). C4d, sC5b-9, and C5a correlated with antiviral antibodies, but not with viral load. Systemic complement activation is associated with respiratory failure in COVID-19 patients and provides a rationale for investigating complement inhibitors in future clinical trials.

## Significance

The new SARS-CoV-2 pandemic leads to COVID-19 with respiratory failure, substantial morbidity, and significant mortality. Overactivation of the innate immune response is postulated to trigger this detrimental process. The complement system is a key player in innate immunity. Despite a few reports of local complement activation, there is a lack of evidence that the degree of systemic complement activation occurs early in COVID-19 patients, and whether this is associated with respiratory failure. This study shows that a number of complement activation products are systemically, consistently, and long-lastingly increased from admission and during the hospital stay. Notably, the terminal sC5b-9 complement complex was associated with respiratory failure. Thus, complement inhibition is an attractive therapeutic approach for treatment of COVD-19.

The ongoing pandemic with the novel severe acute respiratory syndrome coronavirus 2 (SARS-CoV-2) can lead to life-threatening pneumonia and multiple organ failure, termed COVID-19 (1). SARS-CoV-2 infection triggers activation of the innate immune system. It has been hypothesized that a dysregulated innate immune response promotes a phenotype of respiratory failure that may lead to acute respiratory distress syndrome (ARDS) and marked cytokine release (2, 3). Respiratory failure is the main reason for hospital admission and mortality in COVID-19 patients, and new therapeutic interventions are desperately needed (3).

The complement system is a key player in the innate immune response and acts as a danger-sensing alarm system, relying on soluble pattern recognition molecules (4). Complement is activated through three different pathways. The *classical pathway* is triggered by antibodies, but also by acute phase proteins like C-reactive protein (CRP). *Lectin pathway* recognition molecules are mannose-binding lectin (MBL), several ficolins, and collectins. The main function of the *alternative pathway* is to amplify the initial activation from the classical and lectin pathway through the central C3 component, which, in turn, activates C5. Activation of C5 then leads to formation of the potent anaphylatoxin C5a and the terminal C5b-9 complement complex, both exerting proinflammatory actions like recruitment of neutrophils, activation of the adaptive immune system, and endothelial cell activation. By cross-talk with other defense systems like the toll-like receptors and the hemostatic system, the complement system contributes substantially to protection against invading microbes. However, whereas the complement system is important in tissue homeostasis and immune surveillance, overwhelming complement activation may contribute to destructive inflammation harming the host (5, 6).

Complement activation has previously been associated with respiratory failure, ARDS development, and severity in bacterial and viral pneumonia (7, 8). The coronaviruses SARS and Middle East Respiratory Syndrome have both been described to potently induce complement activation, which, in turn, contributes to the development of respiratory failure (9, 10). One preliminary study investigating sC5b-9 and C5a taken within the first week in 31 patients admitted to a critical care unit has shown higher levels in those in need of invasive respiratory therapy (11). Case reports in COVID-19 patients have revealed evidence for deposition of activated complement proteins in lung and other organ tissues (12) in colocalization with COVID-19 spike glycoproteins, hence participating in microvascular injury and thrombosis (13). Indeed, experimental evidence suggests that coronavirus N protein:MASP-2 interaction leads to an uncontrolled activation of the complement lectin pathway (14), and, recently, complement was postulated as a target for therapy in COVID-19 patients (15).

Moreover, the well-established complement inhibitor eculizumab that prevents cleavage of C5, and a neutralizing antibody to C5a, have shown beneficial effect in patient subgroups with COVID-19 (14, 16) in line with one paper showing increased levels of C5a in COVID-19 patients at admission (17). One case was recently treated with the C3 inhibitor compstatin (AMY-101) (18). So far, data on increased systemic complement activation on a broad level of activation products in COVID-19 patients are lacking, and such data will be a prerequisite for complement inhibition as a successful therapeutic approach.

Thus, this study aims to identify the degree and time point of systemic complement activation in COVID-19 patients using a broad spectrum of complement activation products, relate complement activation to clinical course with particular focus on the development of respiratory failure, and thus give a basis for designing clinical trials of therapeutic complement inhibition in COVID-19 patients.

## Results

**Fig. 1.**
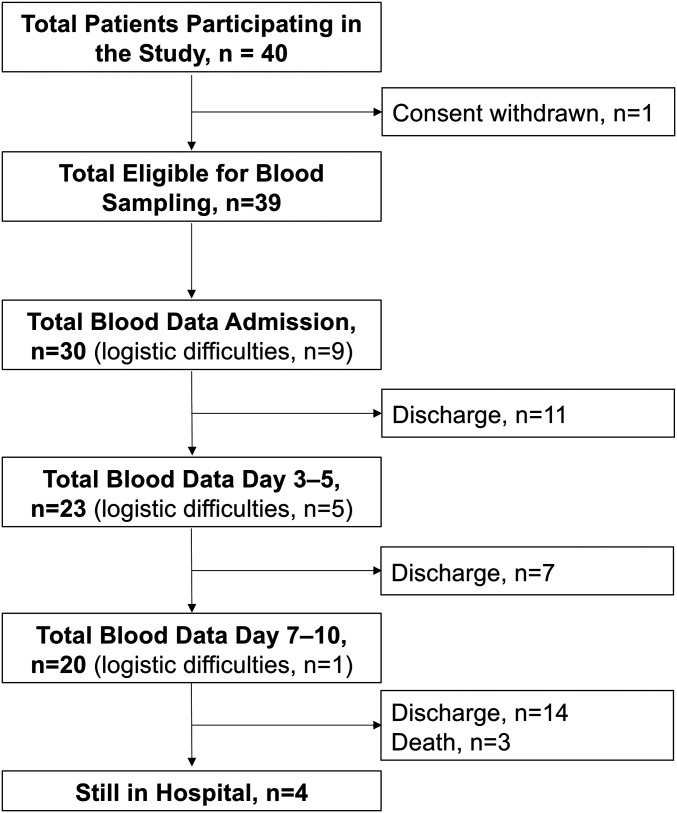
Enrollment and follow-up of patients with COVID-19. Flowchart of inclusion of patients with positive COVID-19 status into the cohort study. Blood sampling to biobank, along with progression of blood data available at each successive time point. Logistic difficulties means that, although patient was available and had consented, no blood was collected at this time point.

Thirty-nine SARS-CoV-2−positive patients were included in the study (Fig. 1). Healthy blood donors served as reference population, with an upper reference limit set at the 95th percentile. Respiratory failure, defined according to the ARDS Berlin definition as PO_2_/FiO_2_ ratio of ≤40 kPa regardless of mechanical ventilatory support, was prominent at admission or developed during hospital stay in 23 patients (59%; Table 1), graded to mild, moderate, and severe in 11, 9, and 3 patients, respectively. Baseline characteristics revealed significant differences in myalgia, fatigue, PO_2_/FiO_2_ ratio, need for oxygen therapy, and Sequential Organ Failure Assessment (SOFA) score between patients with and without respiratory failure (Table 1). Respiratory failure patients showed significantly increased signs of inflammation (white blood cell count and ferritin) and significantly higher fibrin degradation parameter d-dimer at admission (Table 1).

**Table 1.** Demographics, clinical characteristics, and laboratory and radiology findings at admission day

	Total (*n* = 39)	Non-respiratory failure (*n* = 16)	Respiratory failure (*n* = 23)	*P* value
Demographics and clinical characteristics				
Age (years)	61 (49 to 74)	63 (49 to 75)	59 (50 to 70)	0.7
Sex				0.3
Female	10 (25)	6 (38)	4 (17)	
Male	29 (75)	10 (62)	19 (83)	
Comorbidity				
Hypertension	7 (18)	4 (25)	3 (13)	0.3
Diabetes	4 (10)	2 (13)	2 (9)	0.7
Chronic cardiac disease	9 (23)	4 (25)	5 (22)	0.8
Chronic lung disease	9 (23)	5 (31)	4 (17)	0.3
Cancer	2 (5)	2 (13)	0	0.06
Chronic kidney disease	4 (10)	2 (13)	2 (9)	0.7
Symptoms before admission				
Fever (temperature ≥ 37.3 °C)	34 (87)	15 (94)	19 (83)	0.5
Cough	32 (82)	14 (88)	18 (79)	0.5
Sputum	12 (31)	3 (19)	9 (38)	0.2
Sore throat	11 (28)	5 (31)	6 (26)	0.3
Myalgia	21 (54)	11 (69)	10 (44)	***0.04***
Fatigue	30 (77)	11 (69)	19 (83)	***0.03***
Diarrhea	8 (21)	3 (19)	5 (22)	0.3
Nausea or vomiting	15 (39)	6 (38)	9 (39)	0.3
Shortness of breath	23 (59)	7 (44)	16 (70)	0.1
Symptoms and scores at admission				
Need for oxygen therapy	30 (77)	9 (56)	21 (91)	***0.02***
PO_2_/FiO_2_ ratio	39 (30 to 45)	47 (45 to 58)	32 (22 to 39)	***<0.01***
qSOFA score	1 (0 to 1)	0 (0 to 1)	1 (0 to 1)	0.07
SOFA score	2 (1 to 3)	1 (0.5 to 2)	2 (2 to 3)	***0.03***
NEWS score	4.0 (1.8 to 7.0)	3 (1.3 to 5.8)	6 (3.5 to 8)	0.3
Time from first symptom to hospital admission, days	8 (6 to 11)	8 (5 to 11)	9 (7 to 12)	0.6
Laboratory findings				
White blood cell count, x10^9^/L	5.6 (4.8 to 7.7)	4.9 (4.1 to 5.7)	6.6 (5.3 to 9.7)	***<0.01***
Platelet count, x10^9^/L	191 (155 to 231)	180 (150 to 204)	198 (166 to 233)	0.2
D-dimer, mg/L	0.8 (0.6 to 2.0)	0.6 (0.4 to 1.0)	1.0 (0.8 to 2.5)	***0.04***
Ferritin, µg/L	796 (363 to 1355)	498 (267 to 773)	1112 (691 to 1476)	***0.01***
CRP, mg/L	67 (31 to 146)	50 (25 to 76)	114 (44 to 153)	0.05

Data are median (25^th^ to 75th percentile) or n (percent). *P* values were calculated using Mann−Whitney *U* test, χ^2^ test, or Fisher’s exact test, as appropriate. *P* < 0.05 highlighted bold and italic. Respiratory failure is defined as PO_2_/FiO_2_ ratio <40 kPa.

### Complement Activation in Relation to the Degree of Respiratory Failure.

Throughout hospital stay, complement activation markers were above upper reference limit for 74% of assessed samples for sC5b-9, 59% for C5a, 97% for C3bc, 67% for C3bBbP, and 77% for C4d, indicative of long-lasting activation of the complement system (*SI Appendix*, Fig. S1 *A*–*E*). All five complement activation products significantly correlated with each other, suggesting consistent activation of the whole complement system (*SI Appendix*, Fig. S2 *A*–*E*).

**Fig. 2.**
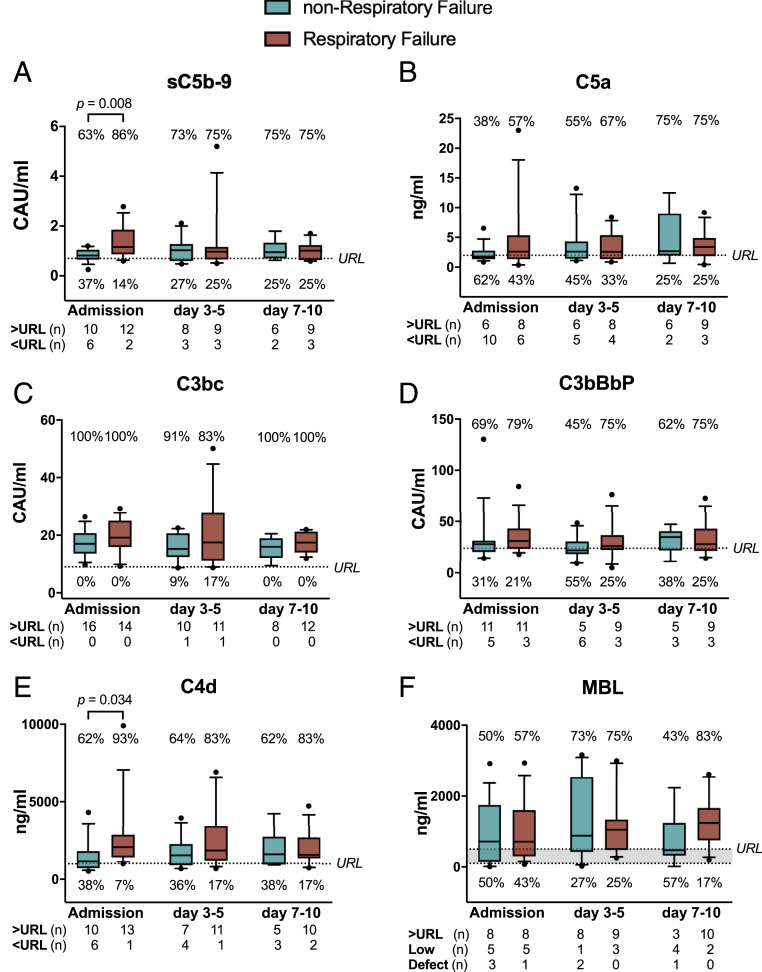
Complement activation products (*A*−*E*) and MBL (*F*) levels in COVID-19 patients with and without respiratory failure at admission and days 3 to 5, and days 7 to 10 of hospitalization. COVID-19 patients were divided according to presence of respiratory failure at day of sampling (defined as PO_2_/FiO_2_ ratio ≤40 kPa). At admission, patients with respiratory failure had significantly higher levels of sC5b-9 (*A*) and C4d (*E*) compared to patients without respiratory failure. The majority of patients had all assessed complement activation products well above the upper reference limits, represented by the dotted lines, at every time point and for every activation product. Results are presented as box plots (line, median; box, interquartile range) with whiskers (10th to 90th percentile). For MBL, the values above the upper dotted line (>500 ng/mL) represent normal values, those between the dotted lines represent low values (100 ng/mL to 500 ng/mL), and those below the lower dotted line represent MBL defects (<100 ng/mL). Mann−Whitney *U* test. URL, upper reference limit; CAU, complement arbitrary units.

Patients with respiratory failure at admission showed significantly higher levels of sC5b-9 and C4d, but not of C5a, C3bc, and C3bBbP, compared to patients with no respiratory failure (Fig. 2 *A*–*E*). The same pattern was observed when including patients who developed respiratory failure during hospital stay, that is, sC5b-9 and C4d were significantly higher at admission in these patients than in patients never experiencing respiratory failure (*P* = 0.03 and *P* = 0.02, respectively). In the mixed model analysis, which included all time points, a significant main effect of daily respiratory failure status on sC5b-9 (F = 5.08, *P* = 0.03) and C4d (F = 5.34, *P* = 0.03) was found, and this effect was dependent on the difference at admission, while it was, at all time points, absent on the other complement activation markers. Likewise, a significant main effect of need for oxygen therapy was found on C4d only (F = 14.24, *P* = 0.001), also dependent on the differences at admission.

### Complement Activation in Relation to the Different Markers of Respiratory Failure.

Regression analysis revealed significant association of sC5b-9 with PO_2_/FiO_2_ ratio (Table 2). Increased sC5b-9 at admission increased odds for daily assessed respiratory failure significantly (OR 31.9 [CI 1.3 to 746.6], *P* = 0.03). Increased C4d at admission significantly increased odds for need for oxygen therapy (11.7 [1.1 to 130.4], *P* = 0.045). Increased sC5b-9 at admission favored increase of respiratory failure severity stage according to ARDS definition in an ordinal regression analysis by 20% (*P* = 0.03). None of the other complement activation products or the recognition molecule MBL were significantly associated with any outcome variables characterizing respiratory failure (Dataset S1).

**Table 2.** Associations between complement and respiratory failure characteristics in COVID-19 patients at hospital admission: Results from linear, logistic, and ordinal regression

Type of model/covariates	R^2^ or OR	95% CI	df or *n*	*P* value
Linear regression				
sC5b-9 vs. PO_2_/FiO_2_ ratio	0.160 R^2^	[−19.12 to −1.154]	29 df	***0.028***
Logistic regression				
sC5b-9 vs. daily RF	31.905 OR	[1.363 to 746.632]	30 *n*	***0.031***
C4d vs. need for oxygen therapy	11.714 OR	[1.052 to 130.421]	27 *n*	***0.045***
Ordinal regression				
sC5b-9 vs. daily RF stage*	0.201 R^2^	[0.132 to 3.047]	30 *n*	***0.033***

RF, respiratory failure (defined as PaO_2_/FiO_2_ ratio ≤40 kPa) registered daily. OR, odds ratio. dF, degrees of freedom. Nagelkerke`s adjusted pseudo R^2^ was used to estimate the coefficient in ordinal regression analyses.

* RF stage according to ARDS Berlin definition (none, mild, moderate, severe).

### Complement Activation in Relation to Markers of Systemic Inflammation, Hemostasis, and Renal Function.

Clinical laboratory markers of inflammation ferritin, CRP, and white blood cell count were significantly correlated with sC5b-9 at admission (*r* = 0.64, *P* < 0.001; *r* = 0.51, *P* = 0.006; *r* = 0.38, *P* = 0.047, respectively). Ferritin at admission was, in addition, significantly correlated with C4d (*r* = 0.69, *P* < 0.001) and C3bc (*r* = 0.53, *P* = 0.005). Platelet count and d-dimer were not significantly correlated with any complement activation product. At admission, sC5b-9, C4d, and C3bc significantly explained 28% (*P* = 0.005), 26% (*P* = 0.007), and 24% (*P* = 0.01) of the variation of ferritin, while only sC5b-9 in addition explained 17% of CRP (*P* = 0.03) in a linear regression analysis (Dataset S1). During the whole time course, only ferritin and CRP were significantly correlated with sC5b-9 and C4d, and CRP correlated also with MBL (*SI Appendix*, Fig. S2*F*). Parameters of renal function, estimated glomerular filtration rate (eGFR) and creatinine, were not correlated with complement activation products either at admission or over the whole time course, except for C3bc and eGFR, which showed a weak, significant association (*r* = 0.25, *P* = 0.03).

### Complement Activation in Relation to Antibodies against SARS-CoV-2.

IgG and IgM against the receptor binding domain (RBD) of the Spike protein were detectable at admission in 60% and 70% of all patients, respectively, and, over time, in 100% and 95% of patients, respectively (*SI Appendix*, Fig. S3). IgG and IgM against the nucleocapsid protein of the CoV were detectable at admission in 70% and 17% of all patients, respectively, and, over time, in 100% and 40% of patients, respectively (*SI Appendix*, Fig. S3). Evidence of a relationship between detectable antibodies against SARS-CoV-2 and complement activation products above upper reference limit was significant for IgG RBD and IgG nucleocapsid protein compared to C4d only (χ^2^ 4.80 and 6.92, *P* = 0.03 and *P* = 0.009, respectively; *SI Appendix*, Table S1). Correlation of absolute antibody and complement activation product levels were significant for IgG nucleocapsid protein and C4d (*r* = 0.30, *P* = 0.01) and IgM nucleocapsid protein with sC5b-9, C4d, and C5a only (*r* = 0.27, 0.32, 0.24; *P* = 0.02, 0.006, 0.03, respectively; *SI Appendix*, Table S2). Antinucleocapsid IgG and IgM levels together with sC5b-9 levels at admission were associated with respiratory failure at admission (OR 37.7 [CI 1.3 to 1,081.9], *P* = 0.03 and 33.8 [1.6 to 689.5], *P* = 0.02, respectively). Antinucleocapsid IgM together with sC5b-9 at admission were associated with respiratory failure during the whole hospital stay (14.4 [1.1 to 184.8], *P* = 0.03) (Dataset S1).

### Complement Activation in Relation to Viral Load.

Samples to correlate viral load with complement activation at admission were available for 21 patients. Viral load ranged from 3.4 to 8.7 (log10 viral RNA copies per mL) with a median of 5.3 (interquartile range 3.8 to 7.3). No significant correlation between viral load and complement activation products was found (*SI Appendix*, Table S3). Notably, viral load at admission was not associated with development of respiratory failure, nor with respiratory failure during the whole hospital stay (Dataset S1).

### MBL Levels in the COVID-19 Cohort.

MBL, the main recognition molecule of the lectin pathway, was very similar to the established levels of MBL in the normal population (Fig. 2*F* and *SI Appendix*, Fig. S2*F*), with a modest increase at days 3 to 5, consistent with an acute phase reaction. Only one patient showed MBL levels below 100 ng/mL at all three time points, consistent with hereditary MBL defect, and this patient did not experience respiratory failure.

### An Example of Extreme In Vivo Complement Activation.

One patient in whom the first and only sample was obtained 7 d after end of the inclusion period and thus 17 d after admission to the hospital, while no clinical data were registered at this time point, was not included in the material and statistics. This patient displayed an extremely high degree of complement activation reflected by all products (see *Materials and Methods* for type of units; here given as the level in this patient [upper reference range and percent increase compared to this in parentheses]): sC5b-9 = 12 (<0.7; 1,714%); C5a = 18 (<2; 900%); C3bc = 80 (<9; 889%); C3bBbP = 94 (<29; 329%); and C4d = 2,536 (<1,000; 253%). At this time point, viral load was in the 25% quartile of the study population (4.9 log10 RNA copies per mL) and IgG antibodies against RBD and nucleocapsid protein present, while IgM were negative. This patient died several days later in a state of multiple organ failure including respiratory failure.

## Discussion

In the present study, a majority of COVID-19 patients showed a consistent increased activation of the complement system, with values above the upper reference limit at hospital admission and throughout the following 10 d. The terminal complement complex sC5b-9 plasma levels were significantly increased in patients with respiratory failure at admission compared to those without respiratory failure. Further, sC5b-9 was associated with daily respiratory failure status.

COVID-19 patients develop virus-related pneumonia, which may lead to life-threatening respiratory failure. The pathophysiology is not fully understood, but SARS-CoV-2 will bind to and cause inflammation in vascular endothelium in general. Increasing evidence suggests that respiratory failure develops due to intraalveolar endothelial damage, resulting in destructive inflammatory responses and terminal complement sC5b-9 deposition in proximity to microvascular injury and thrombosis (13, 19). Increased systemic sC5b-9 and C5a levels have been described in patients admitted to intensive care, while here we show that systemic complement activation is present in the majority of COVID-19 patients admitted to hospital and, importantly, this activation was associated with the presence of respiratory failure at admission or evolvement during hospitalization (11).

Standard clinical parameters of inflammation were correlated with the terminal complement activation product sC5b-9, underscoring the crosstalk between the complement system and other parts on the inflammatory cascade during severe COVID-19 disease (5, 20). A case-series reported antiphospholipid antibodies associated coagulopathy in COVID-19 patients, a state known to induce complement activation that is possible to treat with complement inhibition (21, 22). Thus, complement inhibition was recently suggested as a treatment option for thrombosis in COVID-19 patients (23). The similarity in the pathophysiology of COVID-19 and endotheliopathies like atypical hemolytic uremic syndrome (aHUS) was recently reviewed (24). The fact that these conditions, in particular aHUS, are efficiently treated with a complement inhibitor, has increased the interest for complement therapy in COVID-19 disease.

The long-lasting activation of the complement system with the majority of patients showing levels above clinically accepted reference ranges for days to weeks after hospital admission as observed in this study is of importance. Complement activation products are short-lived and degrade within seconds, like C5a, to 1 h, like sC5b-9. They rapidly return to baseline levels after a single and short-lived stimulus, like extracorporeal circulation or acute trauma (25). Notably, our findings support a state of persistent complement activation in hospitalized COVID-19 patients, which is in-line with findings of increased sC5b-9 and C5a levels in patients admitted to intensive care unit (11). The significance of this prolonged complement activation is uncertain, but one can speculate that it may be caused by antibody production, a continuous viral load with subsequent endothelial, and tissue damage with release of alarmins keeping the complement system activated and inflammation prolonged (1).

We found weak correlations between antibodies and complement activation products in this cohort. Anti-virus IgG displayed a relationship to C4d, only. The classical complement pathway is immediately activated after recognition of danger molecules and is not completely antibody dependent. Only if antibodies are already present after a previous infection or vaccination, antibody binding will initiate classical pathway activation immediately. Thus, while we find some evidence of relationship between anti-virus IgGs and the classical pathway, classical pathway activation could also be due to direct C1 activation since acute phase proteins like CRP can activate C1 directly (26). C4d is generated from the lectin pathway, too, and the lectin pathway is directly activated as first line defense by collectins, ficolins, and MBL, independent of antibodies (27). Thus, we found that antibodies may play a role in the activation of complement in COVID-19 patients. However, this relationship does not seem to be crucial in maintaining the complement response. Hence, antibodies against COVID-19 might represent a double-edged sword; beneficial by neutralizing the virus and detrimental by contributing to over-activation of the complement system as described previously in SARS-CoV infection (28).

SARS-CoV-2 viral load has recently been associated with plasma levels of interferons and cytokines (29). Here, we show that the innate immune response by the complement system at hospital admission is not correlated with viral load. However, hospital admission occurred at a median of 8 d after first disease symptoms, and we therefore cannot exclude that the initial viral load might be associated with complement system activation. The complement system is part of the first-line host defense; that is, it acts upstream and induces a secondary inflammatory response including cytokine production, recruitment of leukocytes, and involvement of the adaptive immune system (4). Therefore, different patterns of association with viral load are explainable and add to the complex picture of immune system activation by SARS-CoV-2.

Likewise, inhibition of the complement system early in the course of an aggravated COVID-19 disease seems reasonable and might be feasible by attenuating the overactivation of the inflammatory system. We suggest that this may be a more powerful strategy than targeting single downstream molecules, like cytokines IL-6 or tumor necrosis factor, which are synthesized later in the course after the immediate immune recognition and activation have occurred (30).

Which specific complement proteins, activation products, or complement receptors should be inhibited is debatable, as inhibitors for many complement targets exist (31). This study reports broad activation of the whole complement system, including C4d from the classical and/or lectin pathway, and C3bBbP from the alternative pathway; thus, specific inactivation of one of the initial pathways seems less likely to result in beneficial clinical effects. As discussed above, whether C4 activation originated from the classical, lectin, or both pathways cannot be concluded from our data.

We measured MBL from the lectin pathway, because it is a very frequent deficiency in the normal population of which 10 to 30% have low levels (32); however, it should be emphasized that the concentration of MBL does not indicate whether the lectin pathway is activated or not. In addition, it has been documented that MBL deficiency increases the risk of SARS-CoV infection (33). In our cohort, MBL levels were comparable to the normal population and even slightly fewer deficiencies were observed than expected (32). Thus, COVID-19 patients have an operative recognition through the lectin pathway, which might be a prerequisite for the described complex formation of the virus’ N protein with MASP-2, one of the serine proteases in the lectin pathway. The anaphylatoxins C3a and C5a and the terminal complement complex C5b-9 are the most potent inducers of inflammation, and inhibition of these should thus be prioritized. Thus, therapeutic inhibition on the level of the common pathway including C3 or C5 inhibition seems most promising in light of the results from this study.

The results in this study are obtained from a small patient cohort, restricting analyses of eventual association between complement activation and survival, as well as generalization of our findings to other cohorts. However, age, sex, and comorbidity distributions are comparable to larger observational cohort studies using the same database registry (34). Thus, these results should be regarded as a hypothesis-generating study for enabling power and sample size estimation for highly desired future clinical studies.

In conclusion, here we show that the complement system is activated systemically in the majority of COVID-19 patients admitted to hospital. All complement activation products were increased, but, especially, the terminal complement sC5b-9 was associated with respiratory failure and systemic inflammation. A hallmark in this patient population is a prolonged and substantial complement system activation. Thus, randomized controlled clinical trials of complement inhibition in COVID-19 patients are justified.

## Materials and Methods

### Study Design and Participants.

#### Patients.

This prospective observational study included two cohorts of adult hospitalized patients (≥18 y old) not included in any randomized clinical trial from Oslo University Hospital and Drammen Hospital, Vestre Viken Hospital Trust, Norway. Informed consent was obtained from all patients’ next-of-kin if the patient was incapacitated to give consent. The SARS-CoV-2 study aiming at virological, clinical, and immunological characterization of COVID-19 was approved by the Regional Ethic Committee (106624, clinicaltrials.gov NCT04381819). As the initial study population, forty consecutive patients in the two hospitals between March 6 and April 14, with acute SARS-CoV-2 infection proven by positive real-time PCR test targeting the E-gene, were eligible for inclusion (35). Due to logistical reasons including high numbers of daily hospital admissions, inclusion of patients was random.

#### Reference population.

Healthy blood donors served as reference populations for the different complement activations assays, as described in detail below.

### Data Collection.

Using a modified version of the International Severe Acute Respiratory and Emerging Infection Consortium/World Health Organization Clinical Characterization Protocol, epidemiological, demographic, clinical, laboratory, treatment, and outcome data were abstracted from electronic medical records into deidentified case record forms on a REDCap database (Research Electronic Data Capture, Vanderbilt University, hosted by University of Oxford). All data were quality-checked by two physicians, and a third researcher adjudicated any difference in interpretation between the two physicians.

### Laboratory Procedures.

Blood samples were obtained at hospital admission (within 48 h), at days 3 to 5, and days 7 to 10 (with one exception when sample was acquired at day 12). Venous blood samples were collected with 4-mL Vacutainer (BD Biosciences) with ethylenediaminetetraacetic acid (EDTA) as anticoagulant. Samples were immediately stored on ice and processed within 30 min, and plasma was isolated by refrigerated centrifugation at 2,000 × *g* for 20 min to obtain platelet-poor plasma and immediately stored at –80 °C.

### Complement Analyses.

All assays were conducted blinded to clinical information. The complement activation products C3bBbP, C3bc, and the terminal complement complex sC5b-9 were quantified using enzyme-linked immunosorbent assays (ELISAs) based on monoclonal antibodies designed against neoepitopes of the products, not reacting with the native component, and performed as described in detail previously (36). The units of these three well-established in-house assays are given according to an international standard defined as complement activation units (CAU) per milliliter with blood donors to define upper reference values of the normal population (36). In detail, 20 females and 20 males aged 18 y to 65 y were analyzed for C3BbBp, C3bc, and sC5b-9 complement activation products. There was no statistical difference between females and males, and the groups were thus combined to 40 individuals. The upper reference limit was set to the 95th percentile, and values above these were regarded as positive. Calculations were made both by absolute values as correlations (Spearman) or as positive versus negative (χ^2^). The commercially available assays were used to measure plasma C4d (SVAR Life Science), C5a, and MBL (Hycult Biotech). The reference values for C4d were given by the supplier, testing EDTA plasma from healthy donors, and, for C5a and MBL, the reference range was based on previously published data from us and others (32, 37).

Summary of the upper reference limits, with the units given above, were for the five activation products: sC5b-9, <0.7; C5a, <2.0; C3bc, < 9.0; C3BbBp, < 24; and C4d, <1,000. For MBL, which is not an activation product but a recognition molecule, the limits are regarded as deficient <100, low in range 100 to 500, and normal >500.

### Antibody Analyses Using Flow Cytometric Bead Assay.

A multiplexed bead-based flow cytometric assay, referred to as microsphere affinity proteomics, was adapted for detection of SARS-CoV-2 antibodies (38, 39). Thus amine-functionalized polymer beads were color-coded with fluorescent dyes as described earlier and reacted successively with amine-reactive biotin (sulfo-NHS-LC-biotin, Proteochem) and neutravidin (Thermo Fisher). A DNA construct encoding the receptor-binding domain of Spike-1 protein (RBD) from SARS-CoV-2 was provided by Florian Krammer, and used to produce recombinant protein as described in *SI Appendix*, *Supplementary Method S1* (40). Bacterially expressed full-length nucleocapsid protein from SARS-CoV-2 was purchased from Prospec Bio. Viral proteins solubilized in phosphate-buffered saline (PBS) were biotinylated chemically using a four to one molar ratio of sulfo-NHS-LC-biotin to protein. Free biotin was removed with G50 sephadex spin columns. Biotinylated proteins were bound to neutravidin-coupled microspheres with fluorescent barcodes and cross-linked with the homobifunctional cross-linker BS3 (Proteochem) for irreversible binding. Beads with Neutravidin only were used as reference for background binding. Sera were diluted 1:1,000 in PBS containing 1% Tween 20 (PBT), 1% bovine serum albumin, 10 ug/mL d-biotin, and 10 ug/mL Neutravidin (Thermo Fisher) and incubated with a mixture of antigen-coupled and Neutravidin-only beads for 1 h at 22 °C under constant agitation. The beads were washed twice in PBT, labeled with R-Phycoerythrin-conjugated goat-anti-Human IgG-Fc or IgM-Fc (Jackson Immunoresearch) for 20 min, washed again, and analyzed by flow cytometry (Attune Next, Thermo Fisher). Specific binding was measured as the ratio of R-Phycoerythrin fluorescence intensity of antigen-coupled beads and neutravidin-only beads. Samples containing antibodies to both nucleocapsid protein and RBD were considered to be positive. A reference panel containing samples from 63 individuals with PCR-confirmed SARS-CoV-2 infection and 86 prepandemic samples was used to set the cutoff. With a cutoff set to obtain a specificity of 100%, the sensitivity was 84%. We also compared antibody concentration detected by flow cytometric analysis to antibodies detected by ELISA (*SI Appendix*, *Supplementary Method S1* and Figs. S4 and S5) and observed good correlation (*SI Appendix*, Fig. S6).

### Viral Load Analyses.

RNA was extracted from clinical samples by MagNA Pure 96 system (Roche). SARS-CoV-2 RNA real-time RT-PCR was performed as described by Corman et al. (35) using E-gene as target. PCR reactions were run on an AriaDx PCR instrument (Agilent Technologies), and viral load was calculated using standard dilution series of purified RNA of SARS-CoV-2 strain Frankfurt1 (European Virus Archive Global). The sample quality was analyzed by RNaseP real-time RT-PCR protocol described by Centers for Disease Control and Prevention (https://www.cdc.gov/coronavirus/2019-ncov/lab/rt-pcr-panel-primer-probes.html; Division of Viral Diseases, National Center for Immunization and Respiratory Diseases), and found valid for all specimens. In cases of PCR inhibition, samples were tested in 1:10 dilutions.

### Outcomes, Calculations, and Statistical Analyses.

In patients missing arterial oxygen tension (PO_2_), PO_2_ was approximated from peripheral O_2_ saturation (41). Likewise, fraction of inspired oxygen (FiO_2_) in nonmechanically ventilated patients was approximated from supplementation of oxygen (42). Respiratory failure was defined according to the pO2/FiO2 ratio limits described in the ARDS Berlin definition regardless of ventilatory support (43). Thus, respiratory failure was present when PO_2_/FiO_2_ ratio was ≤40 kPa. SOFA score was calculated at each time point, while quick SOFA (qSOFA) and National Early Warning Score (NEWS) were calculated at admission day only, due to lack of information on respiratory rate and confusion status at later time points (44). The eGFR was calculated according to the Modification of Diet in Renal Disease formula (45).

Outcome variables for the clinical course of COVID-19 patients were determined before statistical analyses and consisted of dichotomized respiratory failure (yes/no) daily or over the whole hospital course, respiratory failure stage (according to PO_2_/FiO_2_ values used in ARDS classification as none, mild, moderate, and severe), need for oxygen therapy (yes/no), daily FiO_2_, daily PO_2_, and daily PO_2_/FiO_2_ ratio.

Complement activation products were nonnormally distributed and thus transformed using the natural logarithm for comparisons between groups with the linear mixed model analysis with subject as random effect and time and respiratory failure as fixed effects (also as interaction). Further, Mann−Whitney *U*, Χ^2^ test, Spearman´s rank test, and Kruskal−Wallis test were used, as appropriate, for comparison of groups. Linear, logistic, and ordinal regression models were used to assess associations between complement components as predictors of characteristics and stages of respiratory failure (syntax presented in *SI Appendix*, Table S4). More specifically, regression analyses were used to determine risk factors for development of respiratory failure and the composite endpoint of admission to intensive care unit and/or death. The following variables were investigated as independent risk factors: complement activation products, and clinical laboratory markers white blood cell count, ferritin, CRP, d-dimer, and thrombocyte count. Analyses were not corrected for confounders, as sample size was small. Missing values in biobank samples were frequent; thus, number is reported for every analysis. Statistical analyses were performed using IBM SPSS Statistics for Macintosh, Version 26.0 (IBM Corp.) and GraphPad Prism version 8 (GraphPad Software).

## Supplementary Material

Supplementary File

Supplementary File

## Data Availability

Anonymized quantitative data in an SPSS database have been deposited in Norwegian Centre for Research Data and is freely available upon registration for noncommercial use (https://doi.org/10.18712/NSD-NSD2880-V2) (46).
