# Supplementary material for: Systemic complement activation is associated with respiratory failure in COVID-19 hospitalized patients
Source: Proc Natl Acad Sci U S A. 2020 Sep 17;117(40):25018–25. doi: 10.1073/pnas.2010540117 (PMC7547220; doi:10.1073/pnas.2010540117)
Supplement: Supplementary File [file pnas.2010540117.sapp.pdf]

PNAS
www.pnas.org

Supplementary Information for

Systemic Complement Activation is Associated with Respiratory Failure in COVID-19 Hospitalized Patients – A Prospective Cohort Study

Jan C. Holter[a], Soeren E. Pischke[a], Eline de Boer, Andreas Lind, Synne Jenum, Aleksander R. Holten, Kristian Tonby, Andreas Barratt-Due, Marina Sokolova, Camilla Schjalm, Viktoriia Chaban, Anette Kolderup, Trung Tran, Torleif Tollefsrud Gjølberg, Linda G. Skeie, Liv Hesstvedt, Vidar Ormåsen, Børre Fevang, Cathrine Austad, Karl Erik Müller, Cathrine Flateby, Mona Holberg-Petersen, Bente Halvorsen, Fredrik Müller, Pål Aukrust, Susanne Dudman, Thor Ueland, Jan Terje Andersen, Fridtjof Lund-Johansen, Lars Heggelund, Anne M. Dyrhol-Riise, Tom E. Mollnes.
[a] These authors contributed equally to the manuscript

Corresponding author: Soeren E. Pischke
Email: s.e.pischke@medisin.uio.no

This PDF file includes:

- Figures S1 to S3
- Tables S1 to S3
- STROBE checklist
- Supplementary Methods S1 for ELISA method of antibody detection against SARS-CoV-2 including supplemental Figures S4 to S6
- Table S4
- Legend for SI Dataset S1
- Supplementary References

www.pnas.org/cgi/doi/10.1073/pnas.2010540117

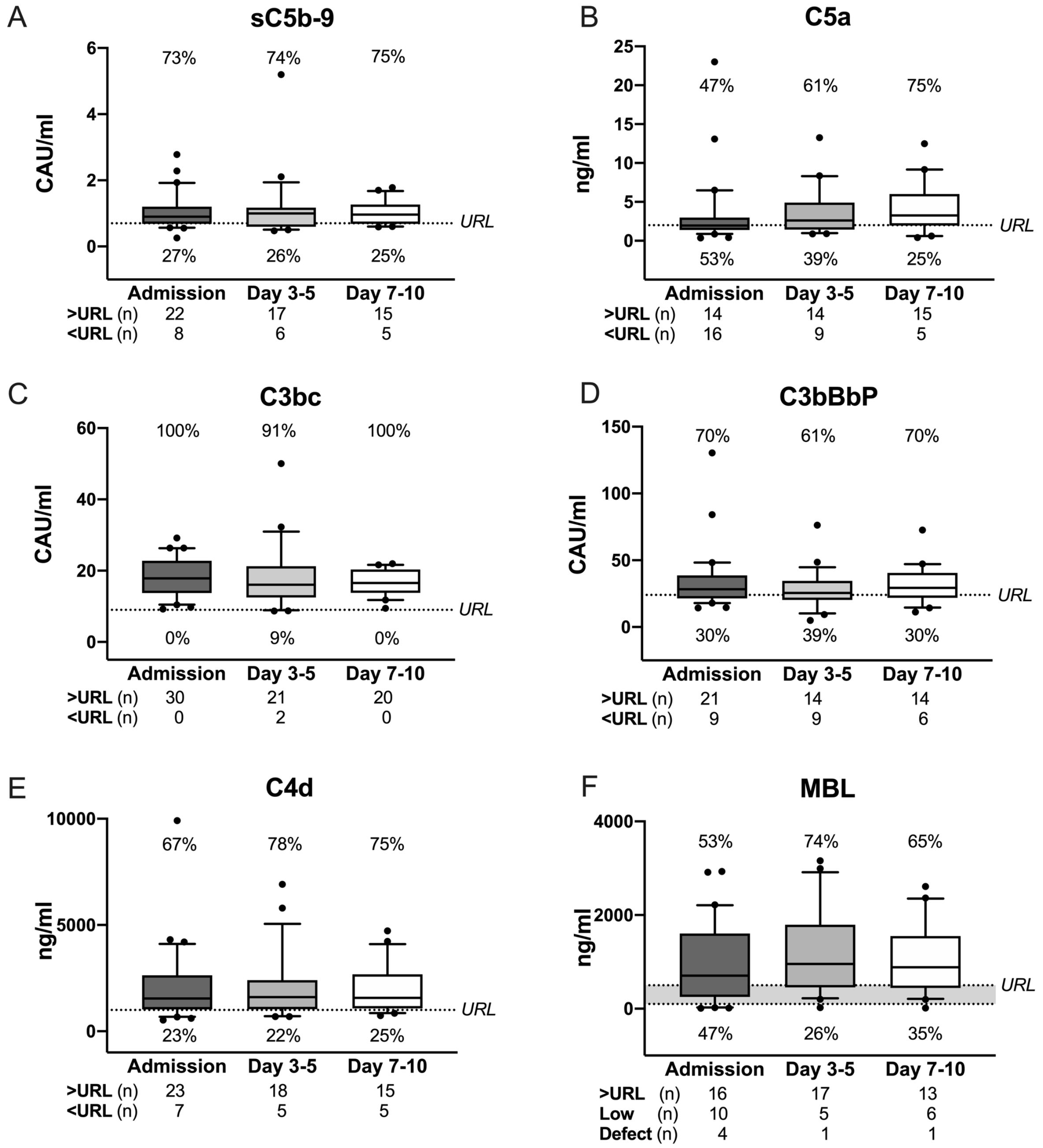

Supplementary Figure S1: Complement activation product levels over time in hospitalized Covid-19 patients.

A-F. All complement activation products were increased above upper reference limit at all time-points, except for C5a at admission. No significant differences were found between time-points. Kruskal-Wallis test with Dunn´s correction test for multiple comparisons.

CAU; complement arbitrary units, URL; upper reference limit, MBL; mannose binding lectin.

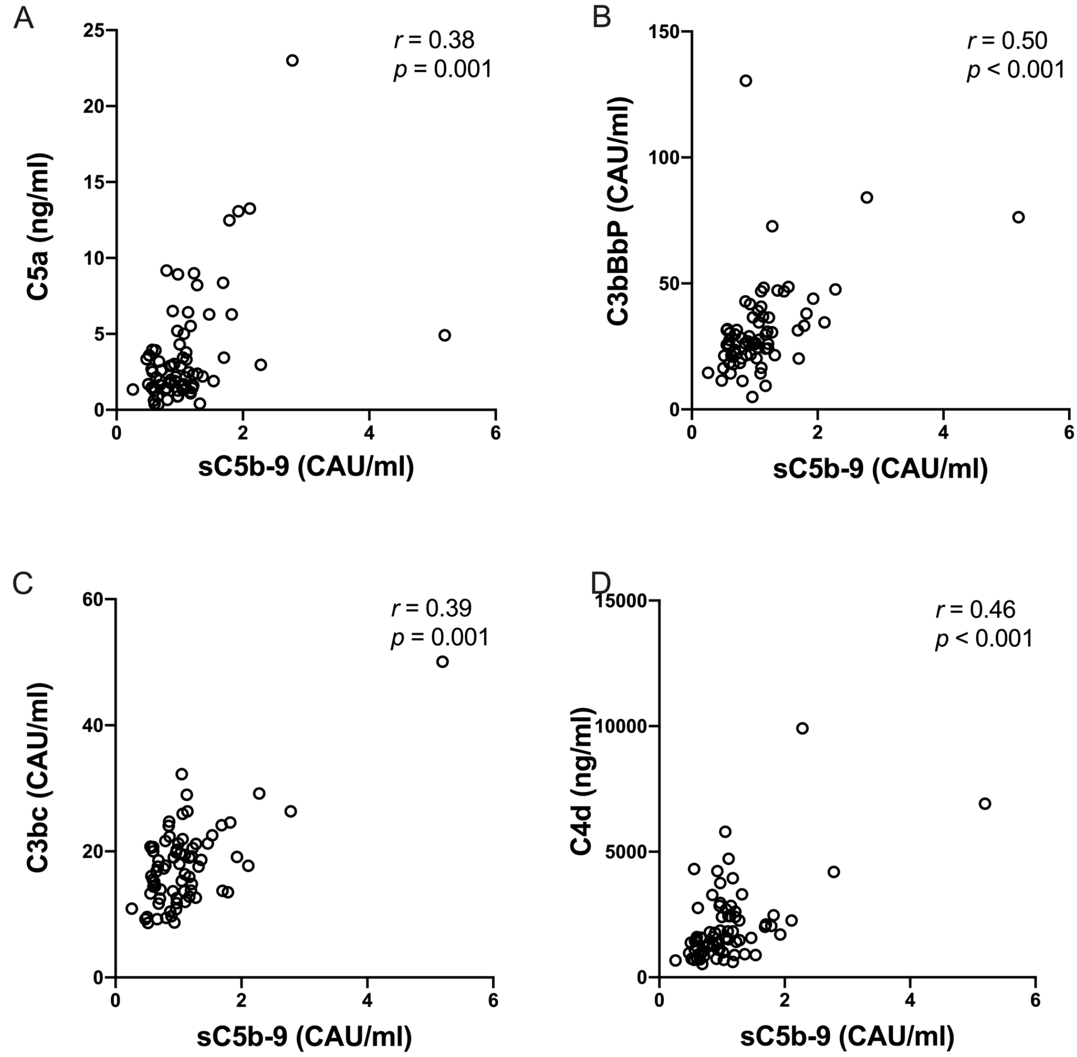

E

	sC5b-9	C5a	C4d	C3bc	C3bBbP	MBL
sC5b-9	1.00					
C5a	0.38 **	1.00				
C4d	0.46 ***	0.29 *	1.00			
C3bc	0.39 **	0.28 *	0.37 **	1.00		
C3bBbP	0.50 ***	0.39 **	0.24 *	0.65 ***	1.00	
MBL	0.05	-0.07	0.11	0.06	-0.05	1.00

* $p < 0.05$
** $p < 0.01$
*** $p < 0.001$

F

	sC5b-9	C5a	C4d	C3bc	C3bBbP	MBL
Ferritin	0.39 **	0.07	0.52 ***	0.22	0.15	0.13
WBC	0.16	-0.04	0.16	-0.04	0.11	0.07
CRP	0.38 *	-0.01	0.29 *	0.13	0.19	0.26 *
Platelets	-0.15	0.08	-0.02	-0.16	0.01	0.09
D-dimer	-0.13	-0.18	0.11	-0.20	-0.26	0.07

Supplementary Figure S2: Correlation among assessed complement proteins and clinical laboratory values of inflammation and hemostasis.

Significant positive correlations were observed between sC5b-9, C5a, C3bBbp, C3bc and C4d levels in Covid-19 patients during hospital stay (A-D). Correlation hemi-matrix with representation of the spearman´s coefficient between all complement activation markers assessed (E) and between complement activation markers and ferritin, white blood cell count, C-reactive protein, platelets and D-dimer. Spearman's correlation. CAU; complement arbitrary units, MBL; mannose binding lectin, WBC; white blood cell count, CRP; C-reactive protein.

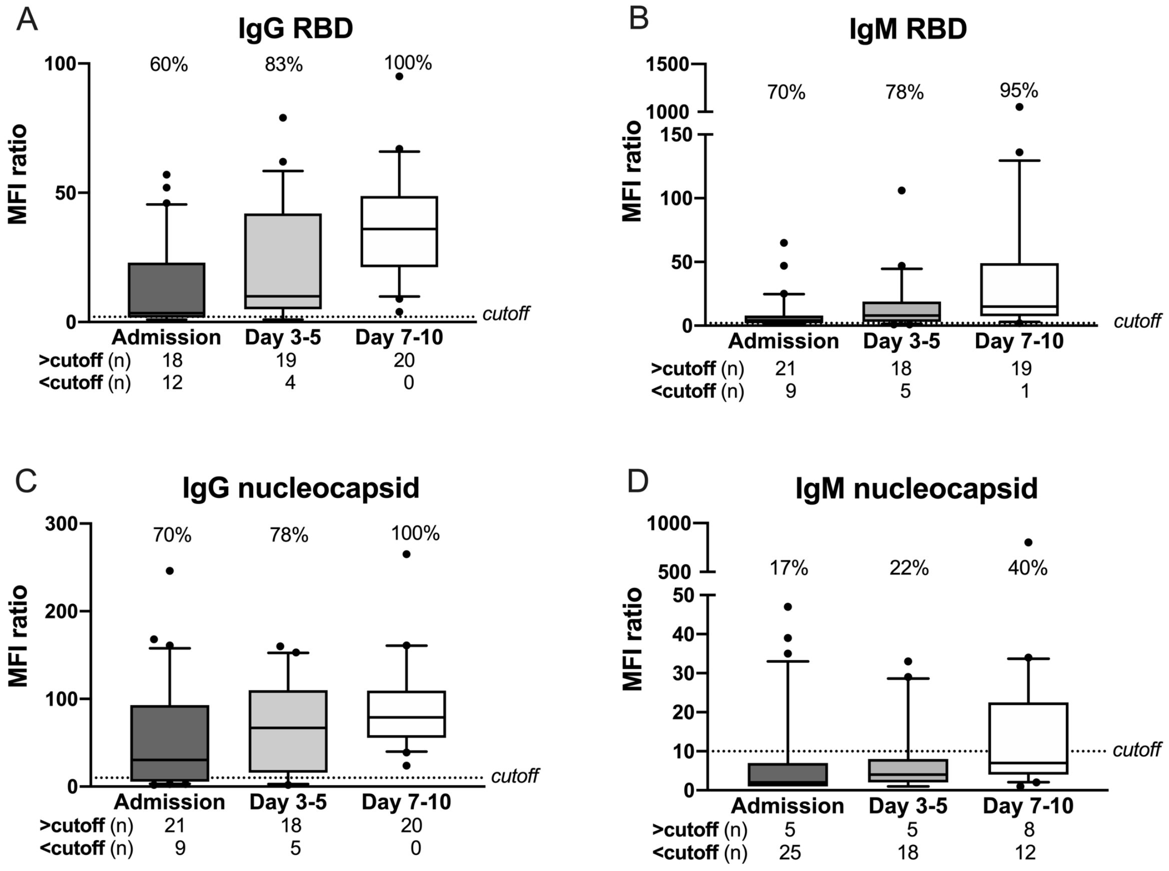

Supplementary Figure S3: Antibodies against SARS-CoV2 proteins over time in hospitalized Covid-19 patients.

A-D. More than 60% of patients showed antibodies against the receptor binding domain of the Spike protein and nucleocapsid at admission with increasing trend throughout hospital stay, except for IgM nucleocapsid where levels remained low. Results are presented as box plots (line; median, box; interquartile range) with whiskers (10 – 90th percentile) and outliers as individual points. The dotted line represents the cutoff determined by pre-pandemic samples. Percent-values represent number of patients with antibodies above cutoff.

Ig; Immunoglobulin, RBD; receptor binding domain of the Spike protein, MFI ratio; mean fluorescence intensity ratio calculated between R-Phycoerythrin fluorescence intensity of antigen-coupled beads and neutravidin-only beads.

Supplementary Table S1. Evidence of relationship between antibodies against SARS-CoV-2 and complement activation products.

	sC5b-9	C4d	C5a	C3bc	C3bBbP
**IgG RBD**	1.40	4.80 *	0.67	0.95	1.85
**IgM RBD**	1.91	3.00	1.17	1.09	1.42
**IgG Nucleocapsid**	0.84	6.92 **	0.57	1.26	1.03
**IgM Nucleocapsid**	1.10	1.98	0.60	0.67	1.23

Ig; Immunoglobulin class G or M, RBD; receptor binding domain.
Reference limits for dichotomization; IgG and IgM RBD > 2, IgG and IgM Nucleocapsid > 10, sC5b-9 > 0.7, C4d > 1000, C5a > 2, C3bc > 9, C3bBbP > 24.
Chi-square test with 1 degree of freedom. Reported is Pearson Chi-square. *; $p < 0.05$ **; $p < 0.01$.

Supplementary Table S2. Correlation matrix of antibodies against SARS-CoV-2 and complement activation products.

	sC5b-9	C4d	C5a	C3bc	C3bBbP	MBL
**IgG RBD**	0.10	0.17	0.01	-0.08	-0.07	0.07
**IgG Nucleocapsid**	0.22	0.30 *	0.22	-0.17	-0.02	-0.13
**IgM RBD**	0.11	0.15	0.02	0.09	0.0	0.04
**IgM Nucleocapsid**	0.27 *	0.32 **	0.25 *	-0.03	0.07	-0.19

Ig; Immunoglobulin class G or M, RBD; receptor binding domain. Spearman correlation. *; $p < 0.05$ **; $p < 0.01$.

Supplementary Table S3. Correlation matrix of viral load and complement activation products at admission.

	sC5b-9	C4d	C5a	C3bc	C3bBbP	MBL
**Viral load**	0.10	-0.23	0.06	-0.17	0.16	0.05

Viral load determined as copies per ml. Spearman correlation. *; $p < 0.05$ **; $p < 0.01$ (no significant correlation detected).

STROBE Statement—Checklist of items that should be included in reports of ***cohort studies***

	Item No	Recommendation
**Title and abstract**	1	(*a*) Indicate the study's design with a commonly used term in the title or the abstract
		(*b*) Provide in the abstract an informative and balanced summary of what was done and what was found
**Introduction**		
Background/rationale	2	Explain the scientific background and rationale for the investigation being reported
Objectives	3	State specific objectives, including any prespecified hypotheses
**Methods**		
Study design	4	Present key elements of study design early in the paper
Setting	5	Describe the setting, locations, and relevant dates, including periods of recruitment, exposure, follow-up, and data collection
Participants	6	(*a*) Give the eligibility criteria, and the sources and methods of selection of participants. Describe methods of follow-up
		(*b*) For matched studies, give matching criteria and number of exposed and unexposed
Variables	7	Clearly define all outcomes, exposures, predictors, potential confounders, and effect modifiers. Give diagnostic criteria, if applicable
Data sources/ measurement	8*	For each variable of interest, give sources of data and details of methods of assessment (measurement). Describe comparability of assessment methods if there is more than one group
Bias	9	Describe any efforts to address potential sources of bias
Study size	10	Explain how the study size was arrived at
Quantitative variables	11	Explain how quantitative variables were handled in the analyses. If applicable, describe which groupings were chosen and why
Statistical methods	12	(*a*) Describe all statistical methods, including those used to control for confounding
		(*b*) Describe any methods used to examine subgroups and interactions
		(*c*) Explain how missing data were addressed
		(*d*) If applicable, explain how loss to follow-up was addressed
		(*e*) Describe any sensitivity analyses
**Results**		
Participants	13*	(a) Report numbers of individuals at each stage of study—eg numbers potentially eligible, examined for eligibility, confirmed eligible, included in the study, completing follow-up, and analysed
		(b) Give reasons for non-participation at each stage
		(c) Consider use of a flow diagram
Descriptive data	14*	(a) Give characteristics of study participants (eg demographic, clinical, social) and information on exposures and potential confounders
		(b) Indicate number of participants with missing data for each variable of interest
		(c) Summarise follow-up time (eg, average and total amount)
Outcome data	15*	Report numbers of outcome events or summary measures over time
Main results	16	(*a*) Give unadjusted estimates and, if applicable, confounder-adjusted estimates and their precision (eg, 95% confidence interval). Make clear which confounders were adjusted for and why they were included
		(*b*) Report category boundaries when continuous variables were categorized
		(*c*) If relevant, consider translating estimates of relative risk into absolute risk for a meaningful time period

Other analyses	17	Report other analyses done—eg analyses of subgroups and interactions, and sensitivity analyses
**Discussion**		
Key results	18	Summarise key results with reference to study objectives
Limitations	19	Discuss limitations of the study, taking into account sources of potential bias or imprecision. Discuss both direction and magnitude of any potential bias
Interpretation	20	Give a cautious overall interpretation of results considering objectives, limitations, multiplicity of analyses, results from similar studies, and other relevant evidence
Generalisability	21	Discuss the generalisability (external validity) of the study results
**Other information**		
Funding	22	Give the source of funding and the role of the funders for the present study and, if applicable, for the original study on which the present article is based

*Give information separately for exposed and unexposed groups.

Note: An Explanation and Elaboration article discusses each checklist item and gives methodological background and published examples of transparent reporting. The STROBE checklist is best used in conjunction with this article (freely available on the Web sites of PLoS Medicine at http://www.plosmedicine.org/, Annals of Internal Medicine at http://www.annals.org/, and Epidemiology at http://www.epidem.com/). Information on the STROBE Initiative is available at http://www.strobe-statement.org.

SUPPLEMENTARY METHOD S1

Production of SARS-CoV-2 RBD. A pCAGGS mammalian expression vector encoding a His-tagged SARS-CoV-2 RBD (1) was transiently transfected into Expi293E™ cells using the Expifectamine™ transfection kit according to the supplier's protocol (Thermo Fisher Scientific). Briefly, the vector and ExpiFectamine™ were diluted in Opti-MEM Reduced Serum Medium (Thermo Fisher Scientific), and added dropwise to the culture with a density of $3*10^6$ viable cells/mL in Expi293 Expression Medium (Thermo Fisher Scientific). Kit enhancers were added 16 hours post-transfection. The transfected cells were kept in a 37 °C incubator with 80% relative humidity and 8% CO_2 on an orbital shaking platform set at 125 rpm for 4 days before medium was harvested by centrifugation at 1200 rpm for 5 minutes.
His-tagged RBD was purified from the collected medium using a HisTrap™ High Performance (GE Healthcare) column and eluted by adding 250 mM imidazole in PBS (pH 7.3). Protein was up-concentrated and buffer exchanged three times to PBS using Amicon® Ultra-4 10K centrifugal filters (Merck Millipore) before determination of the concentration by a DS-11 spectophotometer (DeNovix) and stored at 4°C. Purity was confirmed by SDS-PAGE.

Antibody serology ELISA. 96-well Microtiter wells (Nunc) were coated with 100 µL of 1 µg/mL purified RBD diluted in PBS before incubated overnight at 4°C. The coating solution was then removed and plates blocked with 200 µL PBS containing 4% skimmed milk powder. After incubation for 1 hr at room temperature, blocking solution was removed and plates washed four times with PBS containing 0.05% Tween 20 (PBS/T). Serial dilutions of plasma samples were then added with a starting dilution of 1:50 in PBS/T supplemented with 4% skimmed milk powder (PBS/T/M), 100 µL per well. After incubation for 90 min at room temperature, sample solutions were removed and plates washed four times with PBS/T. ALP-conjugated anti-human IgG MT78 (Mabtech, ref. 3850-9A) was diluted 1:1000, while biotinylated anti-human IgM MT22 (Mabtech, ref. 3880-6-1000) was diluted 1:500 and pre-incubated for 30 min with streptavidin-ALP (Calbiochem, cat. 189732, 1.5 mg/mL) corresponding to a 1:5000 dilution. The anti-IgM antibody and streptavidin-ALP was combined and pre-incubated for 20 minutes before dilution to the correct concentration. All antibodies were diluted in PBS/T/M and 100 µL added to each well followed by incubation for 1 hr at room temperature. Then, plates were washed as above with PBS/T before 100 µL of phosphatase subtrate (Sigma), dissolved in diethanolamine, was added to each well. Absorbance at 405 nm was measured with Tecan Sunrise™ absorbance microplate reader. The data were analyzed in GraphPad Prism 8. The background values (based on pre-COVID plasma as reference) for IgG and IgM were 0.146 (n=21) and 0.398 (n=18), respectively. GraphPad Prism 8 was used to calculate area under curve (AUC) values for all samples without background subtracted.

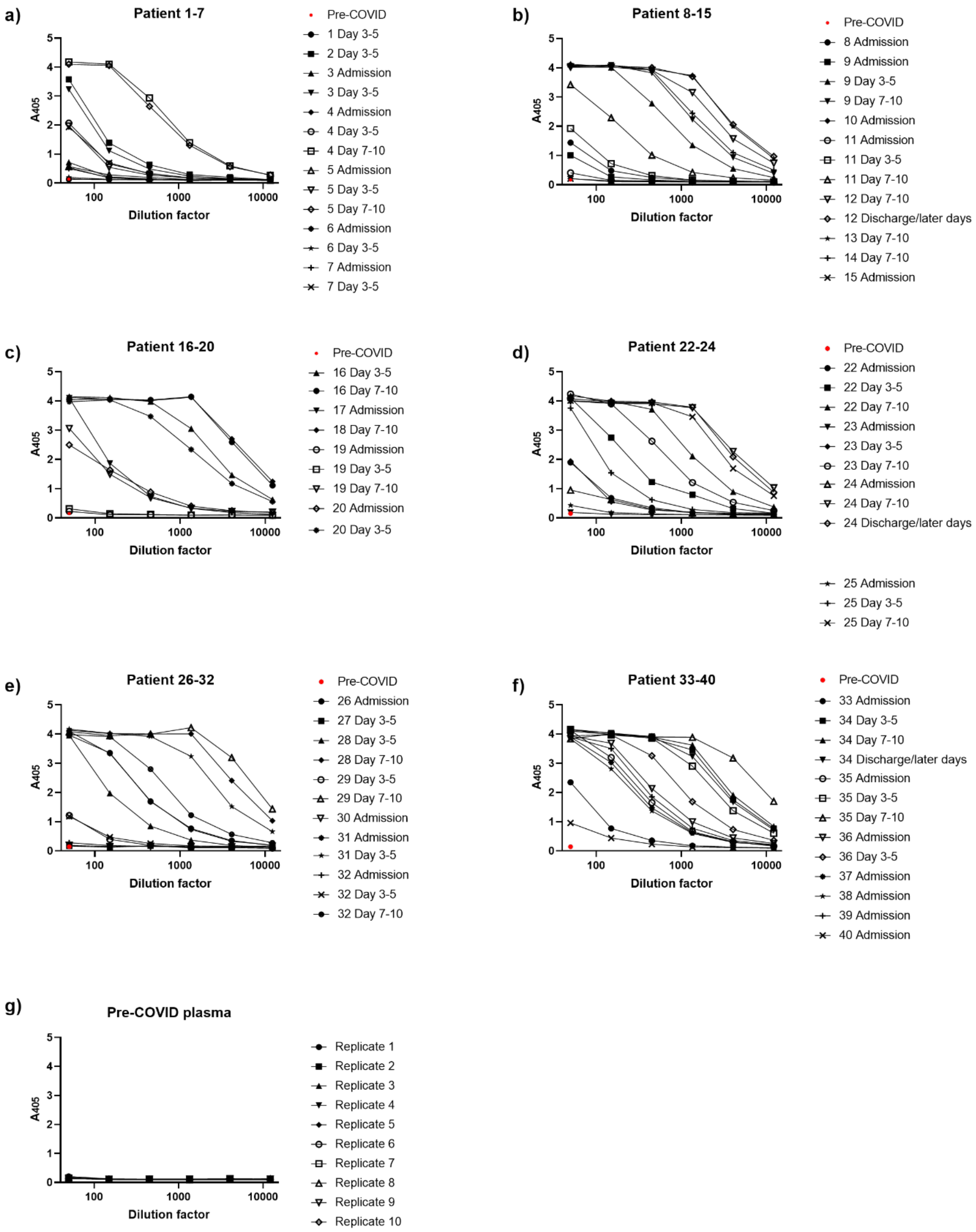

Supplementary Figure S4. Anti-RBD IgG antibody responses.
Binding of serial dilutions of plasma samples to SARS-CoV-2 derived RBD coated in microtiter wells followed by detection with an anti-human IgG antibody. Patient samples taken over several days are displayed in a)-f), shown along with non-titrated pre-COVID-19 plasma taken from a single, healthy donor October 2nd 2019. Ten replicates of the same pre-COVID-19 sample is displayed in g).

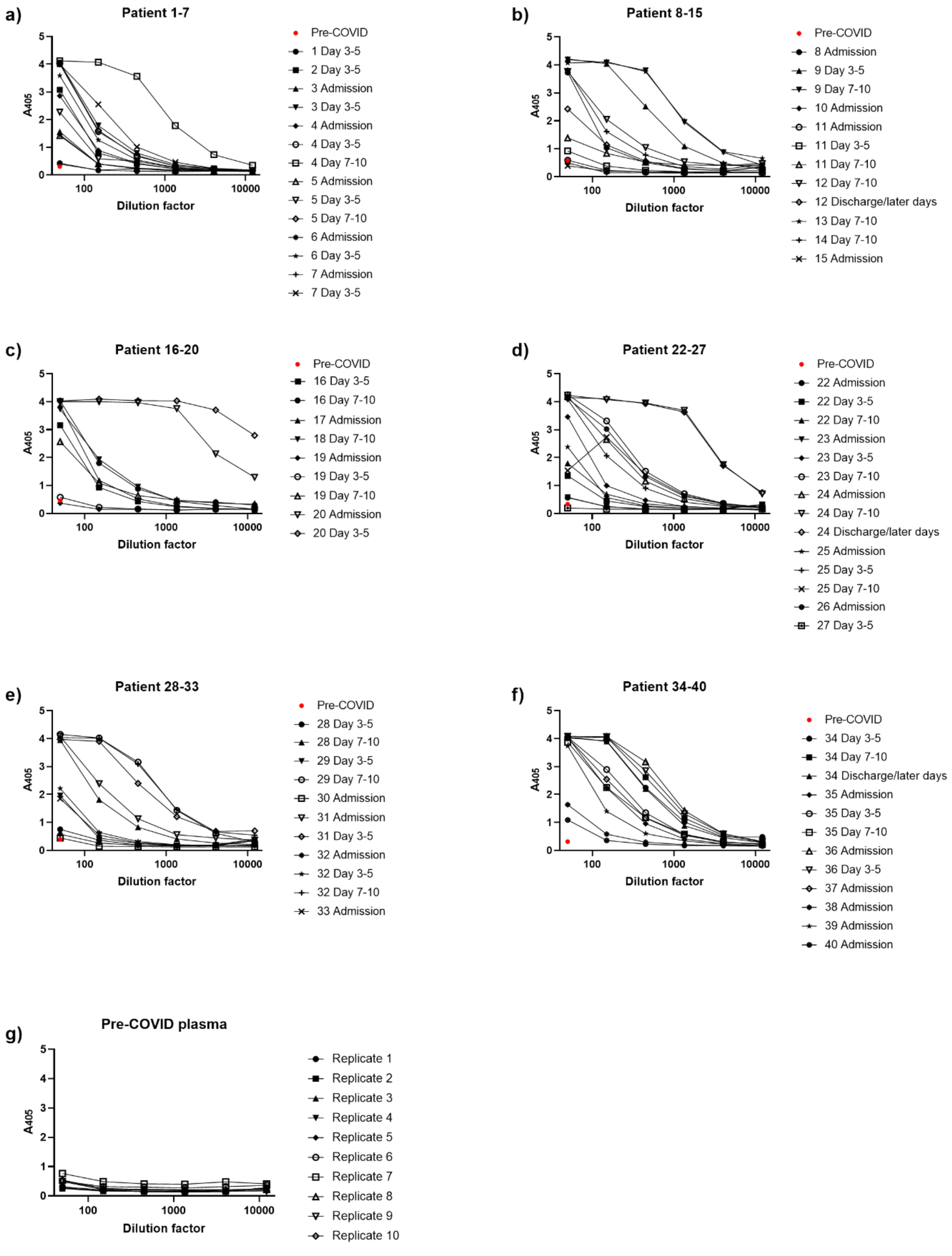

Supplementary Figure S5. Anti-RBD IgM antibody responses.
Binding of serial dilutions of plasma samples to SARS-CoV-2 derived RBD coated in microtiter wells followed by detection with an anti-human IgM antibody. Patient samples taken over several days are displayed in a)-f), shown with non-titrated pre-COVID-19 plasma taken from a single, healthy donor October 2nd 2019. Ten replicates of the same pre-COVID-19 sample is displayed in g).

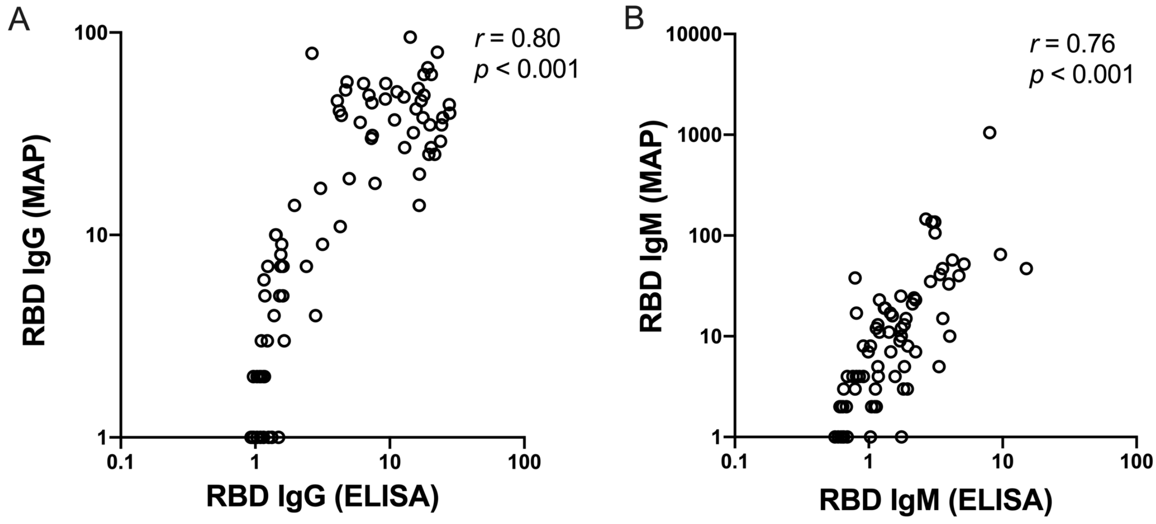

Supplementary Figure S6: Antibody levels detected with microsphere affinity proteomics method correlate with antibody levels detected by ELISA.

Significant positive correlations were observed between IgGs and IgMs against the receptor binding domain of the Spike protein analysed in Covid-19 patients during hospital stay (A,B). Spearman correlation. RBD; receptor binding domain of the Spike protein, Ig; immunoglobin, MAP; microsphere affinity proteomics, ELISA; enzyme linked immunoassay

Supplementary Table S4. Used SPSS syntax for analyzing complement as predictor of respiratory failure characteristics in Covid-19 patients.

Binary outcome variables

LOGISTIC REGRESSION VARIABLES ***Need for oxygen therapy, respiratory failure, ICU stay, invasive ventilation, need for inotropica***
 /METHOD=ENTER ***sC5b-9, C5a, C4d, C3bc, C3bBbP, MBL, PCR_viralload***
 /METHOD=ENTER ***IgG_RBD, IgM_RBD, IgG_Nucleocapsid, IgM_Nucleocapsid***
 /SAVE=COOK RESID SRESID ZRESID
 /PRINT=CI(95)
 /CRITERIA=PIN(0.05) POUT(0.10) ITERATE(20) CUT(0.5).
SUMMARIZE
 /TABLES=COO_ RES_ SRE_ ZRE_
 /FORMAT=LIST NOCASENUM TOTAL LIMIT=100
 /TITLE='Case Summaries'
 /MISSING=VARIABLE
 /CELLS=COUNT.

Continuous outcome variables

REGRESSION
 /MISSING PAIRWISE
 /STATISTICS COEFF OUTS CI(95) R ANOVA CHANGE
 /CRITERIA=PIN(.05) POUT(.10)
 /NOORIGIN
 /DEPENDENT ***PaO_2/FiO_2 ratio, FiO_2, PaO_2, qSOFA, SOFA, NEWS, platelets, d-dimer, ferritin, CRP, WBCs***
 /METHOD=ENTER ***sC5b-9, C5a, C4d, C3bc, C3bBbP, MBL***
 /SCATTERPLOT=(*ZRESID ,*ZPRED)
 /RESIDUALS DURBIN NORMPROB(ZRESID)
 /SAVE COOK RESID ZRESID SRESID.

Complement as continuous outcome variable

REGRESSION
 /MISSING PAIRWISE
 /STATISTICS COEFF OUTS CI(95) R ANOVA CHANGE
 /CRITERIA=PIN(.05) POUT(.10)
 /NOORIGIN
 /DEPENDENT ***sC5b-9, C5a, C4d, C3bc, C3bBbP, MBL***
 /METHOD=ENTER ***PCR_viralload***
 /SCATTERPLOT=(*ZRESID ,*ZPRED)
 /RESIDUALS DURBIN NORMPROB(ZRESID)
 /SAVE COOK RESID ZRESID SRESID.

Ordinal outcome variables

PLUM ***Respiratory failure*** WITH ***sC5b-9, C5a, C4d, C3bc, C3bBbP, MBL***
 /CRITERIA=CIN(95) DELTA(0) LCONVERGE(0) MXITER(100) MXSTEP(5) PCONVERGE(1.0E-6) SINGULAR(1.0E-8)
 /LINK=LOGIT
 /PRINT=FIT PARAMETER SUMMARY TPARALLEL
 /SAVE=ESTPROB PREDCAT PCPROB ACPROB.

Effect of predictors over the whole hospital stay – linear mixed models

MIXED ***natural logarithm of sC5b-9 C5a C4d C3bc C3bBbP MBL*** BY ***Time Predictor (daily respiratory failure status, need for oxygen therapy, or respiratory failure during whole hospital stay)***
 /CRITERIA=DFMETHOD(SATTERTHWAITE) CIN(95) MXITER(100) MXSTEP(10) SCORING(1)
 SINGULAR(0.000000000001) HCONVERGE(0, ABSOLUTE) LCONVERGE(0, ABSOLUTE) PCONVERGE(0.000001, ABSOLUTE)
 /FIXED= ***Time Predictor Time*Predictor*** | SSTYPE(3)
 /METHOD=REML
 /PRINT=COVB SOLUTION TESTCOV
 /RANDOM=INTERCEPT | SUBJECT(***subject***) COVTYPE(VC) SOLUTION
 /REPEATED=***Time*** | SUBJECT(***subject***) COVTYPE(DIAG)
 /EMMEANS=TABLES(***Predictor***) COMPARE ADJ(LSD).

ICU; Intensive Care Unit, RBD; Receptor Binding Domain, qSOFA; Quick Sequential Organ Failure Assessment, NEWS; National Early Warning Score, CRP; C-reactive protein, WBC; white blood cell count.

Legend to SI Dataset S1. Overview of all the results of linear -, logistic - and ordinal regression analyses regarding complement, viral load, antibodies, and respiratory failure (related) characteristics in Covid-19 patients during hospital stay using the syntax displayed in SI Appendix Table S4.

[a]1 outlier was excluded from the analysis
RF; Acute Respiratory Distress Syndrome (defined as PaO_2/FiO_2 ratio ≤40.0 kPa), CI; Confidence interval. Ig; Immunoglobulin, RBD; receptor binding domain of the Spike protein, NC; nucleocapsid protein.
No analyses performed at day 7+ of hospitalization complement versus oxygen therapy because all patients needed oxygen.
Nagelkerke`s adjusted pseudo R-square was used to estimate the coefficient in ordinal regression analyses.

Supplementary Reference

[1] Amanat, F., Stadlbauer, D., Strohmeier, S. *et al.* A serological assay to detect SARS-CoV-2 seroconversion in humans. *Nat Med* (2020). https://doi.org/10.1038/s41591-020-0913-5.
