# Supplementary material for: Systemic complement activation is associated with respiratory failure in COVID-19 hospitalized patients
Source: Proc Natl Acad Sci U S A. 2020 Sep 17;117(40):25018–25. doi: 10.1073/pnas.2010540117 (PMC7547220; doi:10.1073/pnas.2010540117)
Supplement: Supplementary File [file pnas.2010540117.sd01.pdf]

PNAS
www.pnas.org

Supporting Information Dataset for

Systemic Complement Activation is Associated with Respiratory Failure in COVID-19 Hospitalized Patients – A Prospective Cohort Study

Jan C. Holter[a], Soeren E. Pischke[a], Eline de Boer, Andreas Lind, Synne Jenum, Aleksander R. Holten, Kristian Tonby, Andreas Barratt-Due, Marina Sokolova, Camilla Schjalm, Viktoriia Chaban, Anette Kolderup, Trung Tran, Torleif Tollefsrud Gjølberg, Linda G. Skeie, Liv Hesstvedt, Vidar Ormåsen, Børre Fevang, Cathrine Austad, Karl Erik Müller, Cathrine Flateby, Mona Holberg-Petersen, Bente Halvorsen, Fredrik Müller, Pål Aukrust, Susanne Dudman, Thor Ueland, Jan Terje Andersen, Fridtjof Lund-Johansen, Lars Heggelund, Anne M. Dyrhol-Riise, Tom E. Mollnes.
[a] These authors contributed equally to the manuscript

Corresponding author: Soeren E. Pischke
Email: s.e.pischke@medisin.uio.no

This PDF file includes:

Dataset S1

Supporting Information Dataset S1

Type of model / Covariates					
**Linear regression**	**Admission day**	**R-quare**	**95% CI**	**df**	**p value**
**Daily PaO_2/FiO_2 ratio**					
sC5b-9 vs. PaO_2/FiO_2 ratio	3-5	0.044	[-8.414 - 3.122]	21	0.350
sC5b-9 vs. PaO_2/FiO_2 ratio	7+	0.002	[-24.341 - 20.658]	17	0.864
C5a vs. PaO_2/FiO_2 ratio	1	0.057	[-1.872 - 0.416]	29	0.203
C5a vs. PaO_2/FiO_2 ratio	3-5	0.002	[-1.682 - 2.050]	21	0.840
C5a vs. PaO_2/FiO_2 ratio	7+	0.121	[-0.683 - 3.892]	17	0.156
C4d vs. PaO_2/FiO_2 ratio	3-5	0.065	[-0.005 - 0.001]	21	0.252
C4d vs. PaO_2/FiO_2 ratio	7+	0.067	[-0.011 - 0.004]	17	0.300
C3bc vs. PaO_2/FiO_2 ratio	1	0.036	[-1.421 - 0.474]	29	0.315
C3bc vs. PaO_2/FiO_2 ratio	3-5	0.045	[-0.863 - 0.313]	21	0.341
C3bc vs. PaO_2/FiO_2 ratio	7+	0.018	[-2.675 - 1.577]	17	0.592
C3bBbP vs. PaO_2/FiO_2 ratio	1	0.000	[-0.241 - 0.221]	29	0.930
C3bBbP vs. PaO_2/FiO_2 ratio	3-5	0.047	[-0.560 - 0.199]	21	0.333
C3bBbP vs. PaO_2/FiO_2 ratio	7+	0.002	[-0.617 - 0.522]	17	0.862
MBL vs. PaO_2/FiO_2 ratio	1	0.000	[-0.006 - 0.006]	29	0.934
MBL vs. PaO_2/FiO_2 ratio	3-5	0.003	[-0.005 - 0.006]	21	0.874
MBL vs. PaO_2/FiO_2 ratio	7+	0.114	[-0.017 - 0.003]	17	0.171
**Daily FiO_2**					
sC5b-9 vs. daily FiO_2	1	0.075	[-0.022 - 0.148]	29	0.143
sC5b-9 vs. daily FiO_2	3-5	0.095	[-0.015 - 0.087]	22	0.153
sC5b-9 vs. daily FiO_2	7+	0.070	[-0.145 - 0.502]	19	0.261
C5a vs. daily FiO_2	1	0.003	[-0.008 - 0.013]	29	0.620
C5a vs. daily FiO_2	3-5	0.008	[-0.020 - 0.014]	22	0.682
C5a vs. daily FiO_2	7+	0.034	[-0.049 - 0.022]	19	0.433
C4d vs. daily FiO_2	1	0.035	[0.000 - 0.000]	29	0.319
C4d vs. daily FiO_2	3-5	0.145	[0.000 - 0.000]	22	0.073
C4d vs. daily FiO_2	7+	0.054	[0.000 - 0.000]	19	0.323
C3bc vs. daily FiO_2	1	0.036	[-0.004 - 0.013]	30	0.317
C3bc vs. daily FiO_2	3-5	0.082	[-0.002 - 0.009]	22	0.185
C3bc vs. daily FiO_2	7+	0.005	[-0.027 - 0.036]	19	0.768
C3bBbP vs. daily FiO_2	1	0.025	[-0.001 - 0.003]	29	0.404
C3bBbP vs. daily FiO_2	3-5	0.115	[-0.001 - 0.006]	22	0.113
C3bBbP vs. daily FiO_2	7+	0.070	[-0.004 - 0.013]	19	0.259
MBL vs. daily FiO_2	1	0.042	[0.000 - 0.000]	29	0.275

MBL vs. daily FiO_2	3-5	0.054	[0.000 - 0.000]	22	0.284
MBL vs. daily FiO_2	7+	0.053	[0.000 - 0.000]	19	0.330
**Daily PaO_2**					
sC5b-9 vs. daily PaO_2	1	0.014	[-1.765 - 0.941]	29	0.538
sC5b-9 vs. daily PaO_2	3-5	0.007	[-0.998 - 1.423]	21	0.718
sC5b-9 vs. daily PaO_2	7+	0.009	[-1.729 - 2.473]	17	0.712
C5a vs. daily PaO_2	1	0.009	[-0.203 - 0.123]	29	0.617
C5a vs. daily PaO_2	3-5	0.000	[-0.376 - 0.393]	21	0.964
C5a vs. daily PaO_2	7+	0.130	[-0.057 - 0.369]	17	0.141
C4d vs. daily PaO_2	1	0.044	[-0.001 - 0.000]	29	0.267
C4d vs. daily PaO_2	3-5	0.036	[0.000 - 0.001]	21	0.398
C4d vs. daily PaO_2	7+	0.000	[-0.001 - 0.001]	17	0.935
C3bc vs. daily PaO_2	1	0.003	[-0.114 - 0.154]	29	0.763
C3bc vs. daily PaO_2	3-5	0.027	[-0.078 - 0.166]	21	0.462
C3bc vs. daily PaO_2	7+	0.023	[-0.142 - 0.256]	17	0.552
C3bBbP vs. daily PaO_2	1	0.043	[-0.014 - 0.049]	29	0.270
C3bBbP vs. daily PaO_2	3-5	0.017	[-0.053 - 0.095]	21	0.586
C3bBbP vs. daily PaO_2	7+	0.027	[-0.036 - 0.069]	17	0.511
MBL vs. daily PaO_2	1	0.055	[0.000 - 0.001]	29	0.212
MBL vs. daily PaO_2	3-5	0.232	[0.000 - 0.002]	21	***0.023***
MBL vs. daily PaO_2	7+	0.097	[-0.002 - 0.000]	17	0.208
**Quick Sequential Organ Failure Assessment (qSOFA) at admission**					
sC5b-9 vs. qSOFA	1	0.032	[-0.227 - 0.615]	28	0.354
C5a vs. qSOFA	1	0.056	[-0.019 - 0.081]	28	0.217
C4d vs. qSOFA	1	0.090	[0.000 - 0.000]	28	0.113
C3bc vs. qSOFA	1	0.019	[-0.027 - 0.056]	28	0.479
C3bBbP vs. qSOFA	1	0.015	[-0.013 - 0.007]	28	0.529
MBL vs. qSOFA	1	0.053	[0.000 - 0.000]	28	0.229
**Sequential Organ Failure Assessment (SOFA) at admission**					
sC5b-9 vs. SOFA	1	0.008	[-1.344 - 1.946]	20	0.706
C5a vs. SOFA	1	0.000	[-0.207 - 0.190]	20	0.930
C4d vs. SOFA	1	0.015	[-0.001 - 0.000]	20	0.593
C3bc vs. SOFA	1	0.003	[-0.143 - 0.181]	20	0.807
C3bBbP vs. SOFA	1	0.005	[-0.044 - 0.033]	20	0.764
MBL vs. SOFA	1	0.008	[-0.001 - 0.001]	20	0.705
**Daily Sequential Organ Failure Assessment (SOFA)**					
sC5b-9 vs. daily SOFA	1	0.010	[-1.434 - 2.155]	18	0.677
sC5b-9 vs. daily SOFA	3-5	0.255	[-2.722 - 0.173]	12	0.079
sC5b-9 vs. daily SOFA	7+	0.003	[-2.856 - 2.471]	11	0.875

C5a vs. daily SOFA	1	0.002	[-0.234 - 0.199]	18	0.868
C5a vs. daily SOFA	3-5	0.106	[-0.762 - 0.242]	12	0.278
C5a vs. daily SOFA	7+	0.127	[-0.416 - 0.124]	11	0.255
C4d vs. daily SOFA	1	0.000	[-0.001 - 0.001]	18	0.974
C4d vs. daily SOFA	3-5	0.000	[-0.001 - 0.001]	12	0.942
C4d vs. daily SOFA	7+	0.008	[-0.001 - 0.001]	11	0.783
C3bc vs. daily SOFA	1	0.007	[-0.148 - 0.206]	18	0.732
C3bc vs. daily SOFA	3-5	0.302	[-0.285 - -0.001]	12	0.052
C3bc vs. daily SOFA	7+	0.153	[-0.375 - 0.093]	11	0.209
C3bBbP vs. daily SOFA	1	0.012	[-0.049 - 0.035]	18	0.728
C3bBbP vs. daily SOFA	3-5	0.123	[-0.162 - 0.045]	12	0.239
C3bBbP vs. daily SOFA	7+	0.173	[-0.101 - 0.022]	11	0.179
MBL vs. daily SOFA	1	0.005	[-0.001 - 0.001]	18	0.783
MBL vs. daily SOFA	3-5	0.027	[-0.002 - 0.001]	12	0.593
MBL vs. daily SOFA	7+	0.014	[-0.001 - 0.001]	11	0.710
**National Early Warning Score (NEWS) at admission**					
sC5b-9 vs. NEWS	1	0.071	[-0.717 - 4.058]	28	0.163
C5a vs. NEWS	1	0.067	[-0.092 - 0.483]	28	0.175
C4d vs. NEWS	1	0.043	[0.000 - 0.001]	28	0.278
C3bc vs. NEWS	1	0.034	[-0.125 - 0.354]	28	0.336
C3bBbP vs. NEWS	1	0.013	[-0.075 - 0.041]	28	0.553
MBL vs. NEWS	1	0.023	[-0.001 - 0.002]	28	0.427
**Daily platelet count**					
sC5b-9 vs. daily platelet count	1	0.043	[-70.099 - 23.202]	25	0.310
sC5b-9 vs. daily platelet count	3-5	0.016	[-55.183 - 31.668]	21	0.578
sC5b-9 vs. daily platelet count	7+	0.068	[-379.86 - 117.25]	18	0.281
C5a vs. daily platelet count	1	0.005	[-6.638 - 4.799]	25	0.743
C5a vs. daily platelet count	3-5	0.008	[-11.106 - 16.503]	21	0.688
C5a vs. daily platelet count	7+	0.012	[-21.722 - 33.746]	18	0.653
C4d vs. daily platelet count	1	0.023	[-0.019 - 0.009]	25	0.464
C4d vs. daily platelet count	3-5	0.017	[-0.033 - 0.019]	21	0.561
C4d vs. daily platelet count	7+	0.001	[-0.091 - 0.080]	18	0.890
C3bc vs. daily platelet count	1	0.036	[-7.564 - 1.465]	25	0.176
C3bc vs. daily platelet count	3-5	0.000	[-4.631 - 4.300]	21	0.939
C3bc vs. daily platelet count	7+	0.087	[-9.279 - 37.597]	18	0.220
C3bBbP vs. daily platelet count	1	0.002	[-1.004 - 1.243]	25	0.828
C3bBbP vs. daily platelet count	3-5	0.007	[-2.342 - 3.405]	21	0.704
C3bBbP vs. daily platelet count	7+	0.007	[-5.425 - 7.556]	18	0.733
MBL vs. daily platelet count	1	0.036	[-0.042 - 0.016]	25	0.355

MBL vs. daily platelet count	3-5	0.004	[-0.049 - 0.037]	21	0.777
MBL vs. daily platelet count	7+	0.033	[-0.078 - 0.166]	18	0.456
**Daily d-dimer**					
sC5b-9 vs. daily d-dimer	1	0.001	[-1.400 - 3.921]	23	0.591
sC5b-9 vs. daily d-dimer	3-5	0.023	[-7.414 - 4.637]	12	0.622
sC5b-9 vs. daily d-dimer	7+	0.139	[-31.950 - 9.711]	10	0.258
C5a vs. daily d-dimer	1	0.011	[-0.794 - 0.486]	23	0.622
C5a vs. daily d-dimer	3-5	0.058	[-2.576 - 1.170]	12	0.426
C5a vs. daily d-dimer	7+	0.065	[-3.175 - 1.531]	10	0.450
C4d vs. daily d-dimer	1	0.011	[-0.002 - 0.001]	23	0.628
C4d vs. daily d-dimer	3-5	0.042	[-0.005 - 0.002]	12	0.504
C4d vs. daily d-dimer	7+	0.017	[-0.009 - 0.006]	10	0.699
C3bc vs. daily d-dimer	1	0.040	[-0.277 - 0.756]	23	0.346
C3bc vs. daily d-dimer	3-5	0.138	[-0.926 - 0.229]	12	0.211
C3bc vs. daily d-dimer	7+	0.007	[-2.366 - 1.898]	10	0.809
C3bBbP vs. daily d-dimer	1	0.000	[-0.122 - 0.131]	23	0.938
C3bBbP vs. daily d-dimer[a]	3-5	0.001	[-0.058 - 0.052]	11	0.910
C3bBbP vs. daily d-dimer	7+	0.011	[-0.643 - 0.487]	10	0.762
MBL vs. daily d-dimer	1	0.055	[-0.005 - 0.001]	23	0.269
MBL vs. daily d-dimer	3-5	0.079	[-0.008 - 0.003]	12	0.351
MBL vs. daily d-dimer	7+	0.038	[-0.013 - 0.008]	10	0.564
**Daily ferritin**					
sC5b-9 vs. daily ferritin	1	0.279	[259.84 - 1276.47]	26	***0.005***
sC5b-9 vs. daily ferritin	3-5	0.032	[-160.33 - 327.51]	17	0.478
sC5b-9 vs. daily ferritin	7+	0.051	[-547.23 - 1341.2]	16	0.384
C5a vs. daily ferritin	1	0.006	[-58.777 - 84.761]	26	0.712
C5a vs. daily ferritin	3-5	0.021	[-55.962 - 99.368]	17	0.562
C5a vs. daily ferritin	7+	0.001	[-110.973 - 98.98]	16	0.905
C4d vs. daily ferritin[a]	1	0.264	[0.101 - 0.582]	25	***0.007***
C4d vs. daily ferritin	3-5	0.012	[-0.117 - 0.179]	17	0.659
C4d vs. daily ferritin	7+	0.003	[-0.289 - 0.838]	16	0.838
C3bc vs. daily ferritin	1	0.239	[18.615 - 121.408]	26	***0.010***
C3bc vs. daily ferritin	3-5	0.001	[-26.907 - 23.660]	17	0.893
C3bc vs. daily ferritin	7+	0.073	[-43.588 - 134.23]	16	0.294
C3bBbP vs. daily ferritin	1	0.004	[-12.000 - 16.183]	26	0.762
C3bBbP vs. daily ferritin	3-5	0.000	[-16.228 - 16.442]	17	0.989
C3bBbP vs. daily ferritin[a]	7+	0.002	[-20.477 - 17.656]	15	0.876
MBL vs. daily ferritin	1	0.005	[-0.428 - 0.305	26	0.734
MBL vs. daily ferritin	3-5	0.078	[-0.107 - 0.364]	17	0.264

MBL vs. daily ferritin	7+	0.287	[-0.060 - 0.846]	16	*0.027*
**Daily c-reactive protein (CRP)**					
sC5b-9 vs. daily CRP	1	0.166	[6.565 - 128.393]	27	***0.031***
sC5b-9 vs. daily CRP[a]	3-5	0.008	[-74.199 - 109.20]	21	0.695
sC5b-9 vs. daily CRP	7+	0.020	[-152.01 - 269.08]	18	0.565
C5a vs. daily CRP	1	0.000	[-8.353 - 7.682]	27	0.932
C5a vs. daily CRP	3-5	0.006	[-17.347 - 12.345]	22	0.730
C5a vs. daily CRP	7+	0.057	[-33.112 - 11.654]	18	0.326
C4d vs. daily CRP[a]	1	0.033	[-0.013 - 0.034]	26	0.363
C4d vs. daily CRP[a]	3-5	0.019	[-0.021 - 0.039]	21	0.545
C4d vs. daily CRP	7+	0.015	[-0.087 - 0.053]	18	0.615
C3bc vs. daily CRP[a]	1	0.064	[-2.245 - 10.463]	26	0.195
C3bc vs. daily CRP[a]	3-5	0.008	[-4.685 - 6.846]	21	0.700
C3bc vs. daily CRP	7+	0.004	[-22.866 - 17.568]	18	0.786
C3bBbP vs. daily CRP	1	0.003	[-1.370 - 1.772]	27	0.795
C3bBbP vs. daily CRP[a]	3-5	0.063	[-1.582 - 5.565]	21	0.259
C3bBbP vs. daily CRP	7+	0.106	[-1.655 - 8.515]	18	0.173
MBL vs. daily CRP	1	0.002	[-0.037 - 0.045]	27	0.828
MBL vs. daily CRP	3-5	0.035	[-0.026 - 0.065]	22	0.390
MBL vs. daily CRP	7+	0.235	[-0.007 - 0.186]	18	***0.035***
**Daily white blood cell count (WBCs)**					
sC5b-9 vs. daily WBCs	1	0.066	[-0.789 - 3.840]	27	0.187
sC5b-9 vs. daily WBCs[a]	3-5	0.022	[-3.425 - 1.751]	21	0.508
sC5b-9 vs. daily WBCs	7+	0.004	[-2.805 - 5.610]	18	0.491
C5a vs. daily WBCs	1	0.002	[-0.316 - 0.259]	27	0.842
C5a vs. daily WBCs	3-5	0.027	[-0.515 - 0.237]	22	0.450
C5a vs. daily WBCs	7+	0.096	[-0.720 - 0.159]	18	0.196
C4d vs. daily WBCs[a]	1	0.002	[-0.001 - 0.001]	26	0.810
C4d vs. daily WBCs	3-5	0.106	[0.000 - 0.001]	22	0.130
C4d vs. daily WBCs	7+	0.000	[-0.001 - 0.001]	18	0.939
C3bc vs. daily WBCs	1	0.007	[-0.186 - 0.283]	27	0.675
C3bc vs. daily WBCs	3-5	0.044	[-0.063 - 0.177]	22	0.334
C3bc vs. daily WBCs	7+	0.025	[-0.275 - 0.528]	18	0.516
C3bBbP vs. daily WBCs	1	0.009	[-0.043 - 0.069]	27	0.632
C3bBbP vs. daily WBCs	3-5	0.031	[-0.047 - 0.109]	22	0.424
C3bBbP vs. daily WBCs[a]	7+	0.061	[-0.054 - 0.154]	17	0.325
MBL vs. daily WBCs	1	0.002	[-0.001 - 0.002]	27	0.809
MBL vs. daily WBCs	3-5	0.055	[-0.002 - 0.001]	22	0.280
MBL vs. daily WBCs	7+	0.095	[-0.001 - 0.003]	18	0.200

**Viral load (quantified by qPCR) at admission**					
Viral load vs. sC5b-9	1	0.025	[-0.196 - 0.098]	20	0.490
Viral load vs. C5a	1	0.038	[-1.715 - 0.713]	20	0.399
Viral load vs. C4d	1	0.020	[-701.83 - 379.08]	20	0.539
Viral load vs. C3bc	1	0.018	[-2.260 - 0.639]	20	0.256
Viral load vs. C3bBbP	1	0.020	[-8.822 - 4.793]	20	0.543
Viral load vs. MBL	1	0.001	[-246.77 - 211.86]	20	0.875
**Logistic regression**	**Admission day**	**OR**	**95% CI**	**n**	**p value**
**Respiratory failure (RF) during whole stay**					
sC5b-9 vs. RF	1	11.889	[0.913 – 154.756]	30	0.059
sC5b-9 vs. RF	3-5	1.471	[0.374 - 5.790]	23	0.581
sC5b-9 vs. RF	7+	0.769	[0.025 - 23.405]	20	0.880
C5a (per 2 units) vs. RF	1	1.756	[0.722 - 4.272]	30	0.214
C5a (per 2 units) vs. RF	3-5	1.266	[0.623 - 2.572]	23	0.515
C5a (per 2 units) vs. RF	7+	1.057	[0.484 - 2.309]	20	0.889
C4d per 1000 units vs. RF	1	2.135	[0.902 - 5.055]	30	0.085
C4d (per 1000 units) vs. RF	3-5	1.574	[0.659 - 3.761]	23	0.307
C4d (per 1000 units) vs. RF	7+	1.071	[0.327 - 3.513]	20	0.909
C3bc (per 9 units) vs. RF	1	3.531	[0.878 - 14.199]	30	0.076
C3bc (per 9 units) vs. RF	3-5	2.496	[0.594 - 10.498]	23	0.212
C3bc (per 9 units) vs. RF	7+	26.558	[0.387 - 1821.317]	20	0.128
C3bBP (per 24 units) vs. RF	1	1.060	[0.486 – 2.311]	30	0.883
C3bBP (per 24 units) vs. RF	3-5	2.166	[0.335 - 14.000]	23	0.417
C3bBP (per 24 units) vs. RF	7+	1.032	[0.123 - 8.644]	20	0.977
MBL (per 500 units) vs. RF	1	1.055	[0.693 - 1.607]	30	0.801
MBL (per 500 units) vs. RF	3-5	0.914	[0.579 - 1.445]	23	0.702
MBL (per 500 units) vs. RF	7+	1.310	[0.512 - 3.350]	20	0.573
**Daily respiratory failure (RF)**					
sC5b-9 vs. daily RF	3-5	1.422	[0.503 - 4.025]	22	0.507
sC5b-9 vs. daily RF	7+	0.431	[0.030 - 6.273]	18	0.538
C5a (per 2 units) vs. daily RF	1	1.506	[0.777 - 2.918]	30	0.225
C5a (per 2 units) vs. daily RF	3-5	0.900	[0.514 - 1.574]	22	0.711
C5a (per 2 units) vs. daily RF	7+	0.669	[0.362 - 1.236]	18	0.200
C4d (per 1000 units) vs. daily RF	1	1.825	[0.857 - 3.888]	30	0.119
C4d (per 1000 units) vs. daily RF	3-5	1.394	[0.678 - 2.863]	22	0.366
C4d (per 1000 units) vs. daily RF	7+	1.729	[0.535 - 5.588]	18	0.360
C3bc (per 9 units) vs. daily RF	1	2.322	[0.642 - 8.400]	30	0.199
C3bc (per 9 units) vs. daily RF	3-5	1.608	[0.579 - 4.461]	22	0.362

C3bc (per 9 units) vs. daily RF	7+	4.078	[0.268 - 49.094]	18	0.268
C3bBP (per 24 units) vs. daily RF	1	1.132	[0.519 - 2.465]	30	0.756
C3bBP (per 24 units) vs. daily RF	3-5	1.976	[0.404 - 9.657]	22	0.400
C3bBP (per 24 units) vs. daily RF	7+	1.133	[0.218 - 5.880]	18	0.881
MBL (per 500 units) vs. daily RF	1	1.008	[0.663 - 1.531]	30	0.972
MBL (per 500 units) vs. daily RF	3-5	0.808	[0.614 - 1.464]	22	0.808
MBL (per 500 units) vs. daily RF	7+	1.933	[0.832 - 4.495]	18	0.126
**Respiratory failure (RF) moderate and/or severe (m/s) during whole stay**					
sC5b-9 vs. RF m/s	1	1.298	[0.280 - 6.016]	30	0.738
sC5b-9 vs. RF m/s	3-5	1.287	[0.513 - 3.227]	23	0.591
sC5b-9 vs. RF m/s	7+	0.242	[0.017 - 3.494]	20	0.297
C5a (per 2 units) vs. RF m/s	1	0.834	[0.450 - 1.544]	30	0.834
C5a (per 2 units) vs. RF m/s	3-5	0.816	[0.445 - 1.495]	23	0.510
C5a (per 2 units) vs. RF m/s	7+	0.636	[0.333 - 1.217]	20	0.172
C4d (per 1000 units) vs. RF m/s	1	1.438	[0.854 - 2.420]	30	0.172
C4d (per 1000 units) vs. RF m/s	3-5	1.660	[0.834 - 3.303]	23	0.149
C4d (per 1000 units) vs. RF m/s	7+	1.645	[0.653 - 4.144]	20	0.291
C3bc (per 9 units) vs. RF m/s	1	3.318	[0.692 - 15.905]	30	0.134
C3bc (per 9 units) vs. RF m/s	3-5	1.826	[0.687 - 4.856]	23	0.228
C3bc (per 9 units) vs. RF m/s	7+	0.888	[0.105 - 7.475]	20	0.913
C3bBP (per 24 units) vs. RF m/s	1	0.976	[0.386 - 2.466]	30	0.959
C3bBP (per 24 units) vs. RF m/s	3-5	4.008	[0.583 - 27.537]	23	0.158
C3bBP (per 24 units) vs. RF m/s	7+	0.968	[0.214 - 4.371]	20	0.966
MBL (per 500 units) vs. RF m/s	1	1.283	[0.797- 2.067]	30	0.305
MBL (per 500 units) vs. RF m/s	3-5	1.101	[0.714 - 1.697]	23	0.664
MBL (per 500 units) vs. RF m/s	7+	1.156	[0.632 - 2.116]	20	0.637
**Daily respiratory failure (RF) moderate and/or severe (m/s)**					
sC5b-9 vs. daily RF m/s	1	1.802	[0.315 - 10.299]	30	0.508
sC5b-9 vs. daily RF m/s	3-5	1.512	[0.566 - 4.040]	22	0.410
sC5b-9 vs. daily RF m/s	7+	1.929	[0.113 - 33.032]	18	0.650
C5a (per 2 units) vs. daily RF m/s	1	0.332	[0.035 - 3.203]	30	0.341
C5a (per 2 units) vs. daily RF m/s	3-5	0.844	[0.450 - 1.580]	22	0.595
C5a (per 2 units) vs. daily RF m/s	7+	0.608	[0.246 - 1.503]	18	0.281
C4d (per 1000 units) vs. daily RF m/s	1	1.459	[0.881 - 2.416]	30	0.142
C4d (per 1000 units) vs. daily RF m/s	3-5	1.820	[0.785 - 4.219]	22	0.163
C4d (per 1000 units) vs. daily RF m/s	7+	2.029	[0.667 - 6.173]	18	0.213
C3bc (per 9 units) vs. daily RF m/s	1	1.769	[0.295 - 10.607]	30	0.533
C3bc (per 9 units) vs. daily RF m/s	3-5	1.773	[0.645 - 4.869]	22	0.267
C3bc (per 9 units) vs. daily RF m/s	7+	0.364	[0.027 - 4.982]	18	0.449

C3bBbP (per 24 units) vs. daily RF m/s	1	1.164	[0.434 - 3.122]	30	0.763
C3bBbP (per 24 units) vs. daily RF m/s	3-5	3.429	[0.548 - 21.456]	22	0.188
C3bBbP (per 24 units) vs. daily RF m/s	7+	1.053	[0.183 - 6.066]	18	0.954
MBL (per 500 units) vs. daily RF m/s	1	1.214	[0.683 - 2.157]	30	0.509
MBL (per 500 units) vs. daily RF m/s	3-5	1.158	[0.740 - 1.811]	22	0.520
MBL (per 500 units) vs. daily RF m/s	7+	1.354	[0.661 - 2.774]	18	0.408
**Oxygen therapy during whole stay**					
sC5b-9 vs. oxygen therapy	1	2.228	[0.245 - 20.286]	27	0.477
sC5b-9 vs. oxygen therapy	3-5	5.672	[0.192 - 167.554]	20	0.315
C5a (per 2 units) vs. oxygen therapy	1	5.199	[0.543 - 49.796]	27	0.153
C5a (per 2 units) vs. oxygen therapy	3-5	1.409	[0.521 - 3.804]	20	0.499
C4d (per 1000 units) vs. oxygen therapy	3-5	44.917	[0.563 - 3582.843]	20	0.089
C3bc (per 9 units) vs. oxygen therapy	1	3.437	[0.645 - 18.320]	27	0.148
C3bc (per 9 units) vs. oxygen therapy	3-5	3.656	[0.430 - 31.094]	20	0.235
C3bBP (per 24 units) vs. oxygen therapy	1	0.716	[0.318 - 1.615]	27	0.421
C3bBP (per 24 units) vs. oxygen therapy	3-5	0.954	[0.054 - 17.012]	20	0.975
MBL (per 500 units) vs. oxygen therapy	1	1.208	[0.687 - 2.127]	27	0.511
MBL (per 500 units) vs. oxygen therapy	3-5	0.923	[0.568 - 1.868]	20	0.923
**Intensive Care Unit (ICU) during whole stay**					
sC5b-9 vs. ICU stay	1	2.512	[0.507 - 12.441]	30	0.259
sC5b-9 vs. ICU stay	3-5	1.572	[0.594 - 4.158]	23	0.362
sC5b-9 vs. ICU stay	7+	0.944	[0.069 - 12.819]	20	0.965
C5a (per 2 units) vs. ICU stay	1	0.552	[0.123 - 2.471]	30	0.437
C5a (per 2 units) vs. ICU stay	3-5	0.805	[0.401 - 1.616]	23	0.541
C5a (per 2 units) vs. ICU stay	7+	0.707	[0.356 - 1.402]	20	0.321
C4d (per 1000 units) vs. ICU stay	1	1.479	[0.876 - 2.497]	30	0.143
C4d (per 1000 units) vs. ICU stay	3-5	1.543	[0.848 - 2.807]	23	0.155
C4d (per 1000 units) vs. ICU stay	7+	2.408	[0.849 - 6.825]	20	0.098
C3bc (per 9 units) vs. ICU stay	1	3.075	[0.539 - 17.552]	30	0.206
C3bc (per 9 units) vs. ICU stay	3-5	2.425	[0.801 - 7.337]	23	0.117
C3bc (per 9 units) vs. ICU stay	7+	1.033	[0.118 - 9.089]	20	0.976
C3bBP (per 24 units) vs. ICU stay	1	1.094	[0.420 - 2.848]	30	0.854
C3bBP (per 24 units) vs. ICU stay	3-5	2.868	[0.548 - 15.005]	23	0.212
C3bBP (per 24 units) vs. ICU stay	7+	3.863	[0.594 - 25.139]	20	0.157
MBL (per 500 units) vs. ICU stay	1	1.021	[0.586 - 1.778]	30	0.941
MBL (per 500 units) vs. ICU stay	3-5	0.903	[0.554 - 1.469]	23	0.680

MBL (per 500 units) vs. ICU stay	7+	0.926	[0.492 - 1.745]	20	0.813
**Invasive ventilation (IV) during whole stay**					
sC5b-9 vs. IV	1	2.959	[0.547 - 16.016]	30	0.208
sC5b-9 vs. IV	3-5	1.639	[0.619 - 4.344]	23	0.320
sC5b-9 vs. IV	7+	0.945	[0.053 - 16.782]	20	0.969
C5a (per 2 units) vs. IV	1	0.345	[0.037 - 3.205]	30	0.349
C5a (per 2 units) vs. IV	3-5	0.790	[0.371 - 1.684]	23	0.542
C5a (per 2 units) vs. IV	7+	0.794	[0.387 - 1.627]	20	0.528
C4d (per 1000 units) vs. IV	1	1.679	[0.893 - 3.159]	30	0.108
C4d (per 1000 units) vs. IV	3-5	1.157	[0.663 - 2.020]	23	0.608
C4d (per 1000 units) vs. IV	7+	0.813	[0.290 - 2.274]	20	0.693
C3bc (per 9 units) vs. IV	1	4.115	[0.561 - 30.194]	30	0.164
C3bc (per 9 units) vs. IV	3-5	1.682	[0.685 - 4.130]	23	0.257
C3bc (per 9 units) vs. IV	7+	4.286	[0.279 - 65.908]	20	0.297
C3bBP (per 24 units) vs. IV	1	1.208	[0.462 - 3.155]	30	0.700
C3bBP (per 24 units) vs. IV	3-5	2.191	[0.470 - 10.221]	23	0.318
C3bBP (per 24 units) vs. IV	7+	6.010	[0.693 - 52.157]	20	0.104
MBL (per 500 units) vs. IV	1	1.084	[0.600 - 1.959]	30	0.790
MBL (per 500 units) vs. IV	3-5	0.967	[0.589 - 1.588]	23	0.894
MBL (per 500 units) vs. IV	7+	1.111	[0.564 - 2.188]	20	0.762
**Use of inotropica during whole stay**					
sC5b-9 vs. Inotropica	1	0.921	[0.073 - 2.516]	28	0.949
sC5b-9 vs. Inotropica	3-5	0.229	[0.011 - 4.651]	21	0.337
sC5b-9 vs. Inotropica	7+	0.688	[0.023 - 11.925]	19	0.688
C5a (per 2 units) vs. Inotropica	1	0.629	[0.119 - 3.323]	28	0.585
C5a (per 2 units) vs. Inotropica	3-5	0.821	[0.376 - 1.794]	21	0.621
C5a (per 2 units) vs. Inotropica	7+	0.655	[0.276 - 1.552]	19	0.336
C4d (per 1000 units) vs. Inotropica	1	0.477	[0.081 - 2.794]	28	0.412
C4d (per 1000 units) vs. Inotropica	3-5	1.470	[0.684 - 3.159]	21	0.324
C4d (per 1000 units) vs. Inotropica	7+	1.615	[0.647 - 4.028]	19	0.304
C3bc (per 9 units) vs. Inotropica	1	1.980	[0.226 - 17.357]	28	0.537
C3bc (per 9 units) vs. Inotropica	3-5	3.026	[0.667 - 13.715]	21	0.151
C3bc (per 9 units) vs. Inotropica	7+	0.913	[0.075 - 11.537]	19	0.956
C3bBP (per 24 units) vs. Inotropica	1	1.123	[0.368 - 3.427]	28	0.839
C3bBP (per 24 units) vs. Inotropica	3-5	5.967	[0.359 - 99.310]	21	0.213
C3bBP (per 24 units) vs. Inotropica	7+	2.234	[0.394 - 12.658]	19	0.364
MBL (per 500 units) vs. Inotropica	1	1.023	[0.518 - 2.020]	28	0.947
MBL (per 500 units) vs. Inotropica	3-5	0.983	[0.585 - 1.654]	21	0.949
MBL (per 500 units) vs. Inotropica	7+	1.266	[0.631 - 2.540]	19	0.507

**Viral load (quantified by qPCR) at admission**					
Viral load vs. daily RF	1	1.213	[0.585 - 1.654]	25	0.393
Viral load vs. RF during whole stay	1	1.088	[0.698 - 1.696]	25	0.708
**Respiratory failure (RF) during whole stay: adjusted for antibody levels**					
sC5b-9 vs. RF – IgG RBD	1	11.162	[0.821 – 151.685]	30	0.070
sC5b-9 vs. RF – IgM RBD	1	10.976	[0.839 – 143.645]	30	0.068
sC5b-9 vs. RF – IgG NC	1	12.062	[0.889 – 163.732]	30	0.061
sC5b-9 vs. RF – IgM NC	1	14.410	[1.124 – 184.780]	30	***0.040***
C5a (per 2 units) vs. RF – IgG RBD	1	2.074	[0.751 - 5.727]	30	0.159
C5a (per 2 units) vs. RF – IgM RBD	1	1.885	[0.751 - 4.729]	30	0.177
C5a (per 2 units) vs. RF – IgG NC	1	1.829	[0.721 - 4.640]	30	0.204
C5a (per 2 units) vs. RF – IgM NC	1	3.219	[0.903 – 11.474]	30	0.071
C4d per 1000 units vs. RF – IgG RBD	1	2.079	[0.868 - 4.983]	30	0.101
C4d per 1000 units vs. RF – IgM RBD	1	2.202	[0.917 - 5.293]	30	0.078
C4d per 1000 units vs. RF – IgG NC	1	2.105	[0.887 - 4.999]	30	0.092
C4d per 1000 units vs. RF – IgM NC	1	2.185	[0.913 –5.229]	30	0.079
C3bc (per 9 units) vs. RF – IgG RBD	1	3.760	[0.864 - 16.364]	30	0.077
C3bc (per 9 units) vs. RF – IgM RBD	1	4.475	[0.964 - 20.774]	30	0.056
C3bc (per 9 units) vs. RF – IgG NC	1	4.189	[0.940 - 18.677]	30	0.060
C3bc (per 9 units) vs. RF – IgM NC	1	3.390	[0.827 – 13.902]	30	0.090
C3bBP (per 24 units) vs. R–IgG RBD	1	1.072	[0.488 – 2.355]	30	0.863
C3bBP (per 24 units) vs. RF–IgM RBD	1	1.068	[0.49 – 2.332]	30	0.870
C3bBP (per 24 units) vs. RF–IgG NC	1	1.072	[0.489 – 2.347]	30	0.862
C3bBP (per 24 units) vs. RF–IgM NC	1	1.068	[0.487 – 2.344]	30	0.869
MBL (per 500 units) vs. RF–IgG RBD	1	1.074	[0.702 - 1.645]	30	0.742
MBL (per 500 units) vs. RF–IgM RBD	1	1.043	[0.674 - 1.614]	30	0.851
MBL (per 500 units) vs. RF–IgG NC	1	1.076	[0.703 - 1.648]	30	0.735
MBL (per 500 units) vs. RF–IgM NC	1	1.003	[0.645 - 1.558]	30	0.990
**Daily respiratory failure (RF): adjusted for antibody levels**					
sC5b-9 vs. daily RF – IgG RBD	1	31.556	[0.207 - 824.864]	30	0.038
sC5b-9 vs. daily RF – IgM RBD	1	31.951	[1.246 - 819.342]	30	0.036
sC5b-9 vs. daily RF – IgG NC	1	37.744	[1.317 - 1081.91]	30	0.034
sC5b-9 vs. daily RF – IgM NC	1	33.812	[1.658 - 689.476]	30	0.022
C5a (per 2 units) vs. daily RF–IgG RBD	1	1.776	[0.777 - 4.058]	30	0.173
C5a (per 2 units) vs. daily RF–IgM RBD	1	1.624	[0.790 - 3.337]	30	0.187
C5a (per 2 units) vs. daily RF–IgG NC	1	1.570	[0.764 - 3.228]	30	0.220
C5a (per 2 units) vs. daily RF–IgM NC	1	2.078	[0.845 – 5.111]	30	0.111

C4d (per 1000 units) vs. daily RF–IgG RBD	1	1.738	[0.790 - 3.824]	30	0.170
C4d (per 1000 units) vs. daily RF–IgM RBD	1	1.909	[0.860 - 4.240]	30	0.112
C4d (per 1000 units) vs. daily RF–IgG NC	1	1.781	[0.832 - 3.814]	30	0.138
C4d (per 1000 units) vs. daily RF–IgM NC	1	1.836	[0.859 - 3.923]	30	0.117
C3bc (per 9 units) vs. daily RF–IgG RBD	1	2.442	[0.607 - 9.818]	30	0.209
C3bc (per 9 units) vs. daily RF–IgM RBD	1	2.901	[0.693 - 12.137]	30	0.145
C3bc (per 9 units) vs. daily RF–IgG NC	1	2.773	[0.703 - 10.947]	30	0.145
C3bc (per 9 units) vs. daily RF–IgM NC	1	2.269	[0.618 - 8.331]	30	0.217
C3bBP (per 24 units) vs. daily RF–IgG RBD	1	1.155	[0.523 - 2.550]	30	0.722
C3bBP (per 24 units) vs. daily RF–IgM RBD	1	1.145	[0.524 - 2.504]	30	0.734
C3bBP (per 24 units) vs. daily RF–IgG NC	1	1.154	[0.526 - 2.531]	30	0.721
C3bBP (per 24 units) vs. daily RF–IgM NC	1	1.136	[0.520 - 2.484]	30	0.749
MBL (per 500 units) vs. daily RF–IgG RBD	1	1.033	[0.669 - 1.596]	30	0.882
MBL (per 500 units) vs. daily RF–IgM RBD	1	0.990	[0.634 - 1.546]	30	0.966
MBL (per 500 units) vs. daily RF–IgG NC	1	1.039	[0.677 - 1.595]	30	0.861
MBL (per 500 units) vs. daily RF–IgM NC	1	0.978	[0.631 - 1.514]	30	0.919

**Ordinal regression**	**Admission day**	**R-square**	**95% CI**	**n**	**p value**
**Respiratory failure (RF) worst stage during whole stay**					
sC5b-9 vs. RF severity	1	0.086	[-0.374 - 2.223]	30	0.163
sC5b-9 vs. RF severity	3-5	0.011	[-0.618 - 0.979]	23	0.657
sC5b-9 vs. RF severity	7+	0.001	[-2.418 - 2.101]	20	0.891
C5a vs. RF severity	1	0.018	[-0.104 - 0.196]	30	0.547
C5a vs. RF severity	3-5	0.001	[-0.264 - 0.235]	23	0.908
C5a vs. RF severity	7+	0.058	[-0.380 - 0.124]	20	0.321
C4d vs. RF severity	1	0.130	[-0.4×10^{-7} - 0.001]	30	0.080
C4d vs. RF severity	3-5	0.148	[-0.5×10^{-7} - 0.001]	23	0.072
C4d vs. RF severity	7+	0.082	[0.000 - 0.001]	20	0.213
C3bc vs. RF severity	3-5	0.093	[-0.026 - 0.146]	23	0.169

C3bc vs. RF severity	7+	0.007	[-0.174 - 0.258]	20	0.701
C3bBbP vs. RF severity	1	0.000	[-0.029 - 0.031]	30	0.953
C3bBbP vs. RF severity	3-5	0.078	[-0.021 - 0.089]	23	0.222
C3bBbP vs. RF severity	7+	0.003	[-0.049 - 0.065]	20	0.791
MBL vs. RF severity	1	0.013	[-0.001 - 0.001]	30	0.545
MBL vs. RF severity	3-5	0.000	[-0.001 - 0.001]	23	0.983
MBL vs. RF severity	7+	0.056	[-0.000 - 0.002]	20	0.266
**Daily respiratory failure (RF) non, mild, moderate or severe**					
sC5b-9 vs. daily RF stage	3-5	0.027	[-0.536 - 1.117]	22	0.419
sC5b-9 vs. daily RF stage	7+	0.001	[2.171 -2.568]	18	0.869
C5a vs. daily RF stage	1	0.029	[-0.094 - 0.213]	30	0.446
C5a vs. daily RF stage	3-5	0.013	[-0.340 - 0.204]	22	0.622
C5a vs. daily RF stage	7+	0.120	[-0.484 - 0.090]	18	0.178
C4d vs. daily RF stage	3-5	0.064	[0.000 - 0.001]	22	0.282
C4d vs. daily RF stage	7+	0.140	[0.001 - 0.002]	18	0.118
C3bc vs. daily RF stage	1	0.070	[-0.040 - 0.231]	30	0.165
C3bc vs. daily RF stage	3-5	0.374	[-0.049 - 0.131]	22	0.374
C3bc vs. daily RF stage	7+	0.009	[-0.183 - 0.276]	18	0.693
C3bBbP vs. daily RF stage	1	0.005	[-0.025 - 0.036]	30	0.717
C3bBbP vs. daily RF stage	3-5	0.055	[-0.028 - 0.085]	22	0.325
C3bBbP vs. daily RF stage	7+	0.006	[-0.049 - 0.072]	18	0.707
MBL vs. daily RF stage	1	0.002	[-0.001 - 0.001]	30	0.812
MBL vs. daily RF stage	3-5	0.001	[-0.001 - 0.001]	22	0.886
MBL vs. daily RF stage	7+	0.151	[0.000 - 0.002]	18	0.109
